# Prefrontal cortex-to-hypothalamic outputs orchestrate cue-potentiated palatable food consumption via AMPKβ2 signaling

**DOI:** 10.1038/s41421-025-00857-2

**Published:** 2026-01-06

**Authors:** Jiakun Xiang, Minghong Shi, Jiajia Kang, Xingyuan Zhang, Jiankai Ling, Wei Zhan, Dianyi Li, Rongfeng K. Hu, Zhi-Xiang Xu

**Affiliations:** 1 Shanghai Pudong Hospital, Fudan University Pudong Medical Center, State Key Laboratory of Brain Function and Disorders and MOE Frontiers Center for Brain Science, Institutes of Brain Science, Fudan University, Shanghai, China; 2 Institute for Translational Brain Research, State Key Laboratory of Brain Function and Disorders, MOE Frontiers Center for Brain Science, Fudan University, Shanghai, China

**Keywords:** Cell biology, Molecular biology

## Abstract

Cognitive factors critically influence appetite and food consumption, contributing to the increasing incidence of obesity in modern obesogenic environments. However, the cellular and molecular mechanisms underlying this phenomenon remain poorly understood. Here, using calcium imaging in freely moving mice, we found that neurons in the prelimbic cortex (PrL) underwent activity-dependent plasticity in response to learned environmental cues paired with a high-fat diet (HFD). The activity of these neurons reliably predicted the duration of food consumption. Transcriptomic analyses further revealed significant alterations in ATP metabolic processes in the PrL following HFD-associated learning. Notably, the depletion of AMPKβ2, a subunit of AMPK that senses ATP dynamics, abolished PrL plasticity during HFD associative learning and prevented the cue-driven overconsumption of palatable food. At the circuitry level, the activity of PrL^CaMKIIα+^ neuronal projections to orexin neurons in the lateral hypothalamus was required for HFD overconsumption under conditioned contexts. Collectively, our findings elucidate a cellular and molecular framework in a cortical-hypothalamic pathway that regulates cue-evoked HFD overconsumption, highlighting AMPKβ2 as a promising therapeutic target for treating eating disorders.

## Introduction

In mammals, feeding behavior is intricately regulated by an interplay of homeostatic nutrient signals, environmental cues, and cognitive factors^1–10^. Although immediate physiological signals such as hunger and satiety are essential for survival, they are integrated with environmental stimuli, which significantly shape appetite and regulate food intake^11–16^. These interactions are influenced by learned associations, where sensory information from the environment influences the perceived value of stimuli, thereby driving motivated behaviors that promote intake of food, particularly highly palatable and calorie-dense foods^17–21^. Such a learning process involves strengthening neural connections that associate specific cues with food rewards; this is a crucial adaptation mechanism, particularly in resource-scarce contexts, which motivates the search for nutrient-rich foods^18,19,22^. However, in the current obesogenic environment, with readily available high-calorie foods, this adaptive mechanism may inadvertently contribute to the excessive consumption of highly palatable foods that surpass metabolic needs and contribute to the obesity crisis^23–25^. Despite the significant role of environmental contexts in influencing appetite and feeding motivation^26^, the underlying neural substrates, especially cellular and molecular mechanisms, remain to be fully investigated.

At the neural level, the medial prefrontal cortex (mPFC) is suggested to be involved in regulating appetitive drive in both humans and rodents^2,4^. In obese individuals, increased appetitive responsiveness is associated with elevated prefrontal cortex activation^27^, indicating its involvement in feeding behavior. Additionally, the targeted stimulation of the dorsolateral prefrontal cortex has been shown to amplify craving and increase snack food consumption^28^, suggesting that modulating the activity of specific prefrontal regions could offer a strategy for influencing appetite and treating eating disorders. In rodents, immediate early gene expression in the mPFC increases when mice are exposed to food cues^29–31^, and mPFC activity is necessary for food-seeking behavior^32,33^. At the circuitry level, the optogenetic stimulation of dopamine D1 receptor neurons in the mPFC, or their axonal projections to the basolateral amygdala, promotes feeding^34^. Conversely, activating mPFC projections to the nucleus accumbens reduces high-fat diet (HFD) but not chow intake^35^. Furthermore, inhibiting mPFC activity has been shown to alleviate the stress-induced decrease in food consumption but does not alter intake under non-stressful conditions^36^. These mixed findings suggest that the mPFC may regulate food consumption through cellular- and circuitry-specific mechanisms, and that its control over feeding could be influenced by environmental and food palatability cues. However, how mPFC neuronal dynamics encode external and internal feeding cues and how these neural patterns subsequently affect food consumption remain largely unexplored. Moreover, the mPFC is a functionally heterogeneous structure that includes subregions such as the prelimbic and infralimbic cortices, which have been shown to play distinct and even antagonistic roles in learning and memory, decision-making, and cognitive control^37,38^. Nonetheless, the specific functions of these subregions in feeding behaviors remain to be clarified.

AMP-activated protein kinase (AMPK) is an evolutionarily conserved serine/threonine protein kinase; it is activated by increases in AMP-to-ATP and ADP-to-ATP ratios and serves as a master regulator of energy homeostasis^39–43^. AMPK is a heterotrimeric complex comprising catalytic α and regulatory β and γ subunits. The AMPKα subunit contains a kinase domain, where phosphorylation at the threonine residue (Thr172) within the activation loop is pivotal for the catalytic activity of the enzyme^39,41,42^. The AMPKβ subunit serves as a scaffold that enables the assembly of the αβγ complex. Recent studies suggest that AMPKβ also regulates kinase activity through its carbohydrate-binding module, which binds to glycogen and is crucial for stabilizing and activating the AMPK complex across multiple tissues^41,44^. The γ-subunit, with its cystathionine β-synthase domains, binds AMP or ATP, providing the energy-sensing functionality of AMPK. Mammals express multiple isoforms of each subunit, including two α (α1 and α2), two β (β1 and β2), and three γ (γ1, γ2, and γ3) isoforms, which can theoretically form 12 distinct heterotrimeric combinations. However, the specific roles and distribution of these subunits remain to be fully elucidated^40,43,45,46^. Emerging evidence suggests that the tissue expression pattern and isoform-specific roles of AMPK may contribute to the complexity of AMPK function in physiology^39,47^. In the brain, hypothalamic AMPK is considered a key player in the regulation of feeding behavior because of its role in sensing and responding to hormonal and nutritional signals^40,48,49^. Specifically, the phosphorylation of the α subunit at Thr172 activates hypothalamic AMPK and is associated with increased food intake^50,51^. Although the expression of AMPK extends beyond the hypothalamus, including in various brain regions such as the cortex^40^, its functions in these areas, particularly in terms of feeding behavior, are yet to be fully understood.

The excessive intake of palatable, calorie-dense foods triggered by environmental cues contributes to the obesity crisis^23,25^. However, the neural dynamics through which environmental contexts are encoded and prime appetite-responsive neurons and circuits remain elusive. Moreover, the molecular mechanisms that control cue-potentiated overeating have yet to be identified. Here, by employing single cell-resolution miniscope calcium imaging in freely moving mice, we investigated how the neural dynamics of environmental contexts are encoded and activated during HFD consumption bouts. Notably, we found that prelimbic cortex (PrL) neurons exhibit activity-dependent plasticity in response to learned environmental cues paired with an HFD and that their activity can reliably predict the amount of food consumption. Additionally, our data revealed that ATP dynamics play a critical role in the cognitive regulation of feeding behavior, as AMPKβ2 depletion abolished activity-dependent plasticity in PrL neurons during HFD associative learning and prevented the overconsumption of palatable food elicited by environmental cues. Taken together, these findings demonstrate that HFD-paired contexts prime the PrL^CaMKIIα+^→lateral hypothalamus (LH)^orexin+^ circuit to promote HFD consumption and that inhibiting AMPKβ2 could offer a promising strategy for treating eating disorders.

## Results

### PrL^CaMKIIα+^ neurons are activated by HFD consumption and HFD-paired environmental contexts

**Fig. 1:**
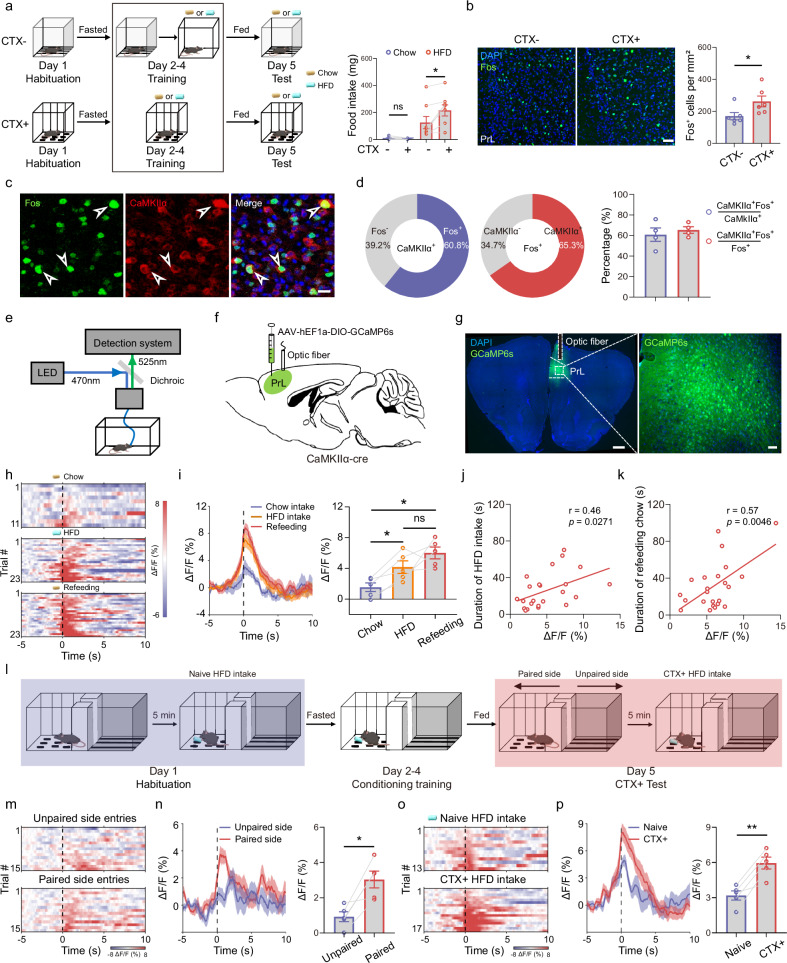
PrL^CaMKIIα+^ neurons are activated by HFD consumption and HFD-paired environmental contexts. **a** Behavioral schematic (left) to examine the effects of environmental cues on food intake. Comparison of chow or HFD intake on the test day in the CTX– and CTX+ groups (right). *n* = 8, Wilcoxon paired test, *P* = 0.25 for chow intake, *P* = 0.016 for HFD intake. **b** Fos staining in the PrL after mice were re-exposed to CTX+ or CTX– contexts. DAPI in blue and Fos in green. Scale bar, 50 μm; *n* = 6, unpaired *t*-test, *P* = 0.045. **c** Representative confocal images showing the colocalization of Fos and CaMKIIα staining in the PrL after re-exposure to the HFD-paired context. Scale bar, 20 μm. **d** Percentage of CaMKIIα^+^Fos^+^ cells among total CaMKIIα^+^ cells or total Fos^+^ cells in the PrL after re-exposure to the HFD-paired context. *n* = 16 sections from 4 mice. **e** Diagram of the fiber photometry recording setup. **f** Schematic of virus injection for the fiber photometry recording of CaMKIIα neurons in the PrL. **g** Representative images showing GCaMP6s expression and the location of the optic fiber in the PrL. Scale bars, 500 μm and 50 μm, respectively. **h** Heatmap showing the response of CaMKIIα neurons in the PrL during chow intake, HFD intake, or chow refeeding after overnight fasting. *n* = 5 mice, 11 trials for chow intake, and 23 trials for HFD intake and chow refeeding; dark dashed line aligned to feeding onset. **i** Left: average response of CaMKIIα neurons in the PrL during feeding behavior. Right: mean ΔF/F during –1–2 s in the chow intake, HFD intake, and chow refeeding trials. *n* = 5 mice; repeated-measures one-way ANOVA with Tukey’s post hoc test, *P* = 0.019 for the comparison of chow and HFD intake, *P* = 0.046 for the comparison of chow intake and refeeding, *P* = 0.436 for the comparison of HFD intake and refeeding. **j** Correlation analysis of mean ΔF/F from –1 s to 2 s during HFD intake in the PrL and HFD intake duration. Pearson correlation analysis, *P* = 0.027. **k** Correlation analysis of mean ΔF/F from –1 s to 2 s during chow refeeding in the PrL and chow refeeding duration. Pearson correlation analysis, *P* = 0.005. **l** Behavioral schematic for the fiber photometry recording of PrL^CaMKIIα^^+^ neurons. **m** Heatmap showing the response of PrL^CaMKIIα+^ neurons to entry into paired and unpaired chambers following conditioning training. Dark dashed line aligned to entry onset; 15 trials from 5 mice. **n** Average response and mean ΔF/F during the 0–2 s interval for PrL^CaMKIIα^^+^ neurons upon entry into paired and unpaired chambers. Dark dashed line aligned to entry onset; *n* = 5 mice, paired *t*-test, *P* = 0.013. **o** Heatmap showing the response of PrL^CaMKIIα^^+^ neurons during naive or CTX + HFD intake. Dark dashed line aligned to feeding onset; 13 trials from 5 mice for naive HFD intake and 17 trials from 5 mice for CTX + HFD intake. **p** Average response and mean ΔF/F during –1–2 s of PrL^CaMKIIα+^ neurons during naive or CTX + HFD intake. Dark dashed line aligned to feeding onset; *n* = 5 mice, paired *t*-test, *P* = 0.006.

Environmental context significantly enhances appetitive drive and food consumption, yet the underlying neural substrates remain poorly understood. We employed a behavioral paradigm adapted from previous studies^13,52^, in which chow or HFD was paired with a contextual chamber (termed CTX+ for the paired feeding training group and CTX– for the non-paired feeding training group) during a three-day training session (Fig. 1a). The intake of either chow or HFD by satiated mice was subsequently measured in a chamber previously associated with the respective diet for 30 min. We found that the context associated with the HFD significantly increased HFD consumption, whereas the chow-paired context had no effect on chow intake (Fig. 1a). This finding prompted us to investigate the neural mechanisms encoding the HFD-paired context, thereby influencing HFD consumption. We re-exposed trained mice to the HFD-paired chamber in the absence of food for 90 min (CTX+ group) and examined the number of Fos^+^ cells in several brain regions implicated in Pavlovian conditioning and feeding behavior, including the prefrontal cortex, hippocampus, amygdala, and hypothalamic nuclei. We observed significantly higher numbers of Fos^+^ cells in the PrL and the central amygdala (CeA) in mice in the conditioned context (CTX+ group) than in the neutral unconditioned context (CTX– group; Fig. 1b and Supplementary Fig. S1a, b). Conversely, there was no change in the number of Fos^+^ cells in several subregions of brain nuclei, including the infralimbic cortex (IL), dentate gyrus (DG), and dorsomedial hypothalamus (DMH), among others (Supplementary Fig. S1a, b). The increased number of Fos^+^ cells in specific brain regions in response to the HFD-paired context suggests their involvement in encoding and retrieving memories linked to conditioned cues, potentially driving appetitive responses to an HFD. We then investigated whether the PrL and CeA respond differently to an HFD than to chow. Compared with chow consumption, a 2-h HFD feeding session significantly increased the number of Fos^+^ cells in the PrL, DG and several hypothalamic subregions, but not in the IL, CeA or CA1 (Supplementary Fig. S1c, d, e). Notably, the number of Fos^+^ cells in the PrL was significantly correlated with the amount of HFD consumption (Supplementary Fig. S1f), indicating that PrL activity is regulated by HFD consumption. Additionally, most neurons activated by HFD feeding or HFD-paired contexts were CaMKIIα^+^ (Fig. 1c, d; Supplementary Fig. S1g, h). Subsequently, an adeno-associated virus (AAV) expressing the genetically encoded calcium indicator GCaMP6s was injected into the PrL to record the bulk calcium dynamics of PrL^CaMKIIα+^ neurons during feeding episodes (Fig. 1e–g). We observed that PrL^CaMKIIα+^ neurons were activated during HFD feeding but exhibited a minimal response during chow feeding. However, these neurons were significantly activated during chow consumption following overnight fasting (Fig. 1h, i). Interestingly, PrL^CaMKIIα+^ neuronal activity increased before both HFD and chow refeeding, peaked during ingestion and was significantly correlated with HFD intake and duration of chow refeeding (Fig. 1i–k), suggesting that the PrL may regulate appetitive motivation influenced by food palatability and nutritional state. Next, we investigated the dynamics of PrL neuronal activity in mice while encountering a conditioned context paired with palatable food. We trained the mice to associate the HFD with one side of a two-chamber box (Fig. 1l, paired side) and found that compared with the entries on the unpaired side, the entries on the paired side in the absence of the HFD post-training activated PrL^CaMKIIα+^ neurons more significantly (Fig. 1m, n). Furthermore, HFD consumption bouts in the paired chamber (CTX + HFD intake) elicited a greater response than HFD intake prior to conditioning training in the context of naive HFD intake (Fig. 1o, p). Taken together, our results indicated that the neural activity of PrL^CaMKIIα+^ neurons is influenced by appetite and associated environmental contexts.

### PrL^CaMKIIα+^ neurons exhibit activity-dependent plasticity in learning to pair environmental contexts with palatable food

**Fig. 2:**
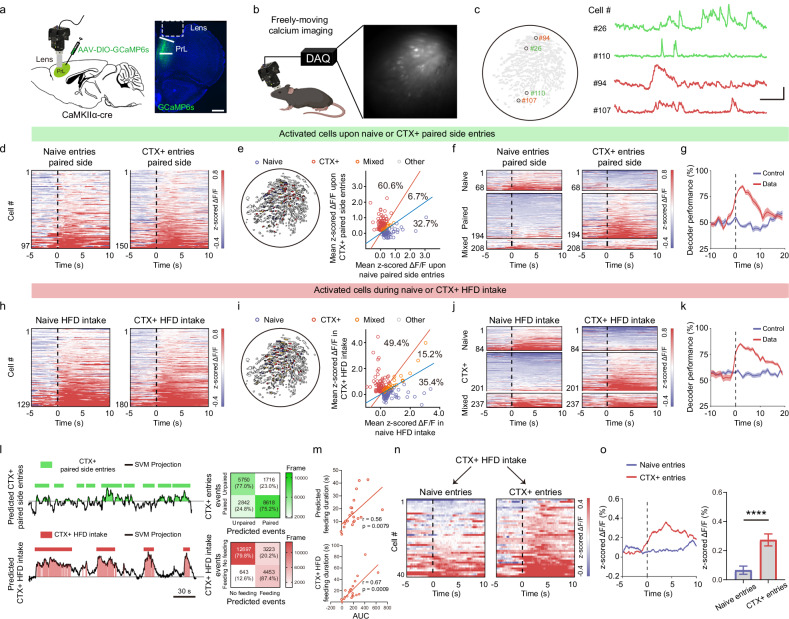
PrL^CaMKIIα+^ neurons exhibit activity-dependent plasticity in learning to pair environmental contexts with palatable food. **a** Diagram of AAV injection and GRIN lens implantation in CaMKIIα-cre mice. Representative image showing the expression of GCaMP6s and the location of the GRIN lens in the PrL. Scale bar, 500 μm. **b** Schematic of miniscope calcium imaging in freely moving mice, with an example of the field of view (FOV) of GCaMP6s-expressing neurons. **c** Representative z-scored ΔF/F Ca^2+^ signal traces of selected neurons within the FOV. Scale bars, 1 z-scored ΔF/F (vertical) and 20 s (horizontal). **d** Heatmap showing the neural activity of PrL^CaMKIIα+^ neurons upon entry into the HFD-paired chambers before (naive) and after (CTX+ ) conditioning training. The dark dashed line is aligned with entry onset. A total of 2068 neurons were recorded from 6 mice for naive entry, and 2175 neurons from 6 mice were recorded for CTX+ entry. **e** FOV example showing the spatial distribution of activated cells during naive or CTX+ paired chamber entry. Cells are classified on the basis of the SRI according to their selective activation by naive or CTX+ entry. **f** Heatmap showing the neural activity of selectively responsive neurons upon naive or CTX+ paired side entry. The dark dashed line is aligned with entry onset. **g** Decoder performance relative to behavior onset in distinguishing between naive and CTX+ paired chambers. **h** Heatmap of the neural activity of PrL^CaMKIIα+^ neurons during HFD consumption bouts in the paired chamber before (naive) and after (CTX+ ) training. The dark dashed line is aligned with the onset of HFD intake. A total of 2068 recorded neurons from 6 mice were used for naive HFD intake, and a total of 2175 recorded cells from 6 mice were used for CTX + HFD intake. **i** FOV example showing the spatial distribution of activated neurons during naive and CTX + HFD consumption in the paired chamber. These activated neurons were categorized as selectively responsive to naive and CTX + HFD intake on the basis of their SRI. **j** Heatmap showing the neural activity of selectively responsive neurons, categorized by SRI, during naive and CTX + HFD consumption. The dark dashed line is aligned with the onset of HFD intake. **k** Decoder performance relative to behavior onset in distinguishing between naive and CTX + HFD intake. **l** Left: Decoder prediction of each behavior using the population activity of neurons activated during CTX+ paired-side entry (top) and CTX + HFD intake (bottom). The plots show projections of population activity onto the SVM hyperplane, and the light patches show decoder predictions. Scale bar, 30 s. Right: comparison of the matrix between predicted events by activated cells in CTX+ paired-side entry and CTX + HFD intake vs corresponding actual observed events. **m** Correlation analysis between the area under curve (AUC) of neural activity of CTX + HFD intake neurons and both the predicted feeding duration (top) and the actual observed feeding duration (bottom). 21 trials from 6 mice; Spearman correlation analysis for actual observed feeding, *P* = 0.0009; Pearson correlation analysis for predicted feeding, *P* = 0.0079. **n** Heatmap showing the neural activity of the same CTX + HFD intake neurons upon entry into the paired chamber before (naive) and after (CTX+ ) conditioning training. The dark dashed line is aligned with entry onset. **o** Response of CTX + HFD intake neurons upon entry into the paired chamber before (naive) and after (CTX+ ) conditioning training. The dark dashed line is aligned with entry onset. The bar graph shows the mean z-scored ΔF/F from 0 s to 5 s after entry. Wilcoxon matched-pairs signed rank test, ****P < 0.0001.

Bulk calcium fiber photometry revealed that conditioned environmental contexts enhanced the activity of PrL^CaMKIIα+^ neurons during HFD consumption. To determine whether the increased neural activity results from a shift in neural ensembles or an increase in the strength of existing neuronal responses, we employed single-cell miniscope calcium imaging to investigate the neural dynamics of PrL^CaMKIIα+^ neurons exposed to conditioned environmental contexts and palatable food (Fig. 2a–c). After pairing contextual chambers with the HFD, we found that the percentage of activated PrL^CaMKIIα+^ neurons tended to increase upon entering the HFD-paired chamber (termed CTX+ neurons) compared with entering the same side of the chamber before training sessions (naive neurons; Supplementary Fig. S2a). Interestingly, when we computed the selectivity response index (SRI; see Materials and Methods) on the basis of neuronal activation by naive or CTX+ chamber entries, we found that a substantial portion of the neurons activated in these two conditions did not overlap (Fig. 2d, e), indicating that the PrL differentially encodes HFD-paired vs neutral contexts. Furthermore, linear support vector machine (SVM) decoders^53^ trained on the activity of PrL^CaMKIIα+^ neurons differentiated between HFD-paired and neutral contexts, with decoding accuracy significantly greater than that on shuffled data and peaking 3 s after entering the chamber (Fig. 2f, g; Supplementary Fig. S2b, c). These findings indicate that PrL^CaMKIIα+^ neurons reliably encode HFD-paired and neutral contexts at the population level. Mice consumed more of the HFD in the HFD-paired chamber than in the neutral context, prompting us to investigate whether unique PrL neural dynamics are engaged during HFD feeding after conditioned training. Remarkably, although the percentage of PrL^CaMKIIα+^ neurons activated during HFD consumption was comparable in neutral and paired contexts (termed naive and CTX + HFD intake neurons, respectively; Supplementary Fig. S2d), we found that the neurons activated by HFD feeding in these contexts were mostly distinct, with only 15.2% of the neurons being activated across both conditions. The two groups of neurons were anatomically intermixed in the PrL without evident spatial clustering (Fig. 2h, i). Furthermore, neural responses during HFD feeding episodes in the HFD-paired and neutral contexts could be decoded at the population level (Fig. 2j, k; Supplementary Fig. S2e, f), suggesting that neural responses nonetheless recruited largely separate populations of PrL^CaMKIIα+^ neurons and were associated with distinct population codes. Next, we explored whether the amount of HFD consumption could be predicted by the activation pattern of PrL^CaMKIIα+^ neurons. Using linear decoders, we found that entering or HFD intake in the conditioned chamber could each be predicted from the population activity of PrL^CaMKIIα+^ neurons with an accuracy above 75% (Fig. 2l), suggesting that each behavior is robustly encoded at the population level. More importantly, both the feeding duration predicted by the SVM model and the actual observed feeding duration were notably correlated with the activity of CTX + HFD intake neurons (Fig. 2m), suggesting a causal link between the elevated neural activity of PrL^CaMKIIα+^ neurons and increased HFD consumption in conditioned environmental contexts.

The observation that HFD intake in conditioned and neutral contexts elicits distinct population activity raises further questions about how environmental contexts are encoded and retrieved during HFD feeding episodes and how they influence subsequent HFD consumption. Given that the duration of HFD consumption can be predicted from the activity of PrL^CaMKIIα+^ neurons (Fig. 2l, m), we hypothesized that the activity of these neurons undergo activity-dependent plasticity post-training and that CTX + HFD intake neurons are activated upon exposure to conditioned environmental cues, enhancing appetitive drive and HFD consumption. Indeed, when we computed the z-scored responses of the same CTX + HFD intake neurons upon entry into the paired chamber before and after conditioning training, we observed a significant increase in their activity after training compared with before training (Fig. 2n, o). These findings suggest that these neurons undergo activity-dependent plasticity, elevating their neural activity in response to a conditioned context. Additionally, the activity-dependent plasticity observed in CTX + HFD intake neurons is specifically related to conditioned environmental contexts, rather than changes in intrinsic activity properties during conditioning training, as indicated by the significantly low z-scored responses of these neurons during HFD consumption in a home cage environment post-training (Supplementary Fig. S2g, h).

The above results demonstrate that PrL neurons responsive to HFD intake exhibit plasticity changes according to environmental contexts. We subsequently explored the neural dynamics of PrL neurons encoding conditioned environmental contexts during HFD-feeding episodes and how these dynamics vary with different states of conditioning learning. We found that 46% of the CTX+ neurons were activated when the HFD was consumed in the conditioned chamber (Supplementary Fig. S2i), indicating substantial shared activation of the neuronal encoding of conditioned environmental cues and HFD consumption. These co-activated neurons could be categorized into two groups: one group activated ~5 s before HFD feeding bouts (pre-cells), and the other group activated during the consummatory phase (post-cells) (Supplementary Fig. S2j). Interestingly, both the number (from 15.3% to 28%) and the average responses of pre-cells increased upon entry into the paired chamber after conditioned training (Supplementary Fig. S2i–k), suggesting a potential role in promoting appetite drive toward an HFD elicited by conditioned cues. In contrast, there was no obvious change in the number of post-cells (15.3% vs 18%) before and after conditioning learning (Supplementary Fig. S2i–l).

Collectively, the single-cell calcium imaging results revealed a close interaction between neurons encoding environmental contexts and those responsive to HFD consumption. The learned associations between environmental contexts and palatable food enhance the activity of PrL^CaMKIIα+^ neurons through activity-dependent plasticity, and the activity of these neurons predicts the amount of HFD consumption upon re-exposure to conditioned environmental contexts.

### PrL^CaMKIIα+^ neurons bidirectionally regulate HFD consumption in an HFD-paired context

**Fig. 3:**
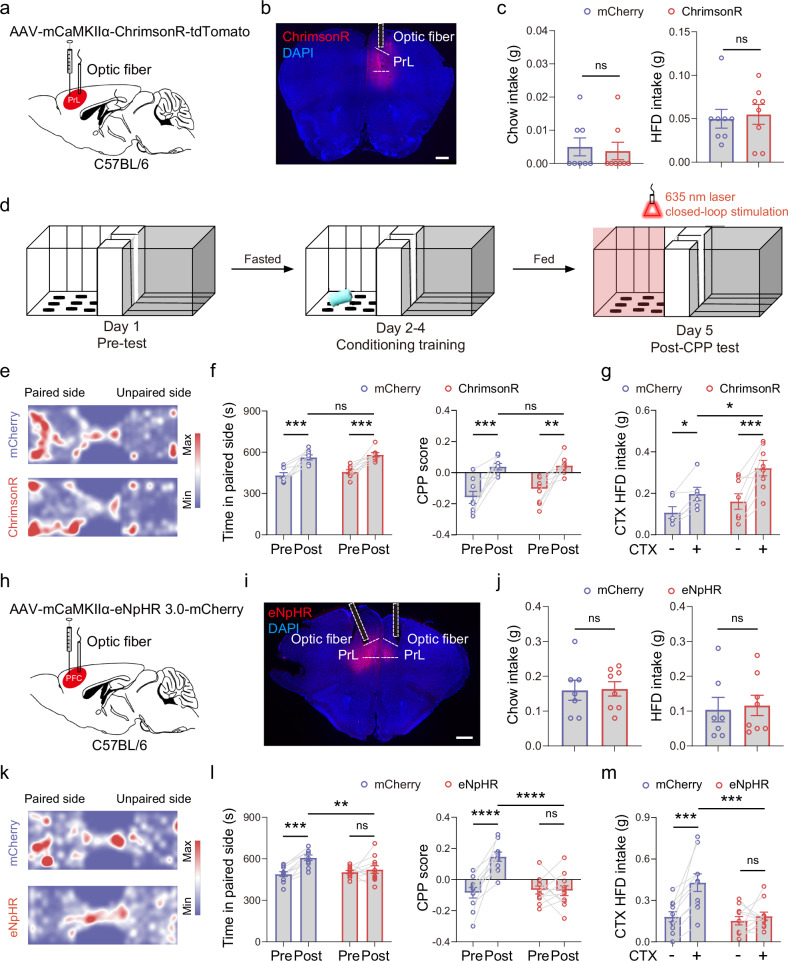
PrL^CaMKIIα+^ neurons bidirectionally regulate HFD consumption in HFD-paired contexts. **a** Schematic of virus injection and optic fiber implantation for the optogenetic stimulation of PrL^CaMKIIα+^ neurons. **b** Representative image showing the expression of ChrimsonR and the location of the optic fiber in the PrL. Scale bar, 500 μm. **c** Chow or HFD intake with the optogenetic activation of PrL^CaMKIIα+^ neurons; 8 mice per group. **d** Behavioral schematic for examining place preference for HFD-paired environmental contexts following conditioning training with the closed-loop optogenetic stimulation of PrL^CaMKIIα+^ neurons. **e** Heatmaps representing the position in the experimental arena during the CPP test for mice expressing mCherry or ChrimsonR in the PrL. **f** Time spent on the paired side before and after paired training and the CPP scores for mice expressing mCherry or ChrimsonR. Repeated-measures two-way ANOVA followed by Sidak’s multiple comparison post hoc test. F_(1, 14)_ = 54.08, *P* < 0.0001 for time; F_(1,14)_ = 40.16, *P* < 0.0001 for CPP scores; 8 mice per group. **g** HFD intake in the CTX– and CTX+ chambers measured in mice expressing mCherry or ChrimsonR in the PrL. Repeated-measures two-way ANOVA followed by Sidak’s multiple comparison post hoc test. F_(1, 12)_ = 29.09, *P* = 0.0002 for contexts; mCherry, 6 mice; ChrimsonR, 8 mice. **h** Schematic of virus injection and optic fiber implantation for the optogenetic inhibition of PrL^CaMKIIα+^ neurons. **i** Representative image showing the expression of eNpHR and the location of the optic fiber in the PrL. Scale bar, 500 μm. **j** Measurement of chow intake in fasted mice or HFD intake in sated mice with the optogenetic inhibition of PrL^CaMKIIα+^ neurons. mCherry, 7 mice; eNpHR, 8 mice. **k** Heatmaps representing the position in the experimental arena during the CPP test for mice expressing mCherry or eNpHR in the PrL. **l** Time spent on the paired side before and after paired training and CPP scores for mice expressing mCherry or eNpHR. Repeated-measures two-way ANOVA followed by Sidak’s multiple comparison post hoc test. F_(1, 19)_ = 7.686, *P* = 0.012 for interaction in time comparison; F_(1, 19)_ = 9.970, *P* = 0.005 for CPP scores. mCherry, 10 mice; eNpHR, 11 mice. **m** HFD intake in the CTX+ and CTX– chambers measured in mice expressing mCherry or eNpHR in the PrL with continuous optogenetic inhibition. Repeated-measures two-way ANOVA followed by Sidak’s multiple comparison post hoc test. F_(1, 19)_ = 8.319, *P* = 0.0095 for the virus. *P < 0.05, **P < 0.01, ***P < 0.01, ****P < 0.0001, ns not significant. mCherry, 10 mice; eNpHR, 11 mice.

The above results demonstrated that PrL^CaMKIIα+^ neurons are activated upon exposure to an HFD-paired context and selectively respond to an HFD rather than chow. Next, we applied optogenetics to investigate whether PrL neural activity regulates feeding. We injected an AAV encoding ChrimsonR under the mouse CaMKIIα promoter into the PrL, allowing PrL^CaMKIIα+^ neuron activation upon light stimulation (Fig. 3a, b). Surprisingly, although PrL neurons were activated during HFD consumption (Fig. 1h, i; Supplementary Fig. S1c–e), the optogenetic activation of PrL^CaMKIIα+^ neurons did not significantly alter either chow or HFD intake (Fig. 3c). However, after pairing contextual chambers with chow or the HFD (Fig. 3d; Supplementary Fig. S3a), we found that, compared with mice expressing mCherry, those receiving optogenetic stimulation of PrL^CaMKIIα+^ neurons expressing ChrimsonR significantly increased HFD consumption in the paired chamber (Fig. 3g). In contrast, this stimulation did not affect chow consumption (Supplementary Fig. S3d). The differential regulation of chow and HFD intake is unlikely to be attributed to place preference for the paired chambers, as mice spent significantly more time in these chambers under both conditions (Fig. 3e, f; Supplementary Fig. S3b, c). Additionally, in a real-time place preference test under neutral conditions, ChrimsonR- and mCherry-expressing mice spent comparable amounts of time on the optogenetically stimulated side (Supplementary Fig. S3e). We then bilaterally injected an AAV encoding the light-driven chloride pump halorhodopsin eNpHR into the PrL (Fig. 3h, i). The subsequent inhibition of PrL^CaMKIIα+^ neurons did not affect chow intake in fasted mice or HFD intake in sated mice (Fig. 3j). However, it prevented place preference for chambers paired with the HFD and blocked HFD overconsumption in the paired chamber (Fig. 3k–m). The blocked preference effect was not caused by changes in overall locomotor activity between eNpHR- and mCherry-expressing mice (Supplementary Fig. S3f). Together, our findings indicate that PrL^CaMKIIα+^ neuronal activity is crucial for establishing a place preference linked to food contexts and can bidirectionally regulate HFD overconsumption triggered by environmental contexts.

### AMPKβ2 signaling in the PrL influences cue-potentiated HFD overconsumption

**Fig. 4:**
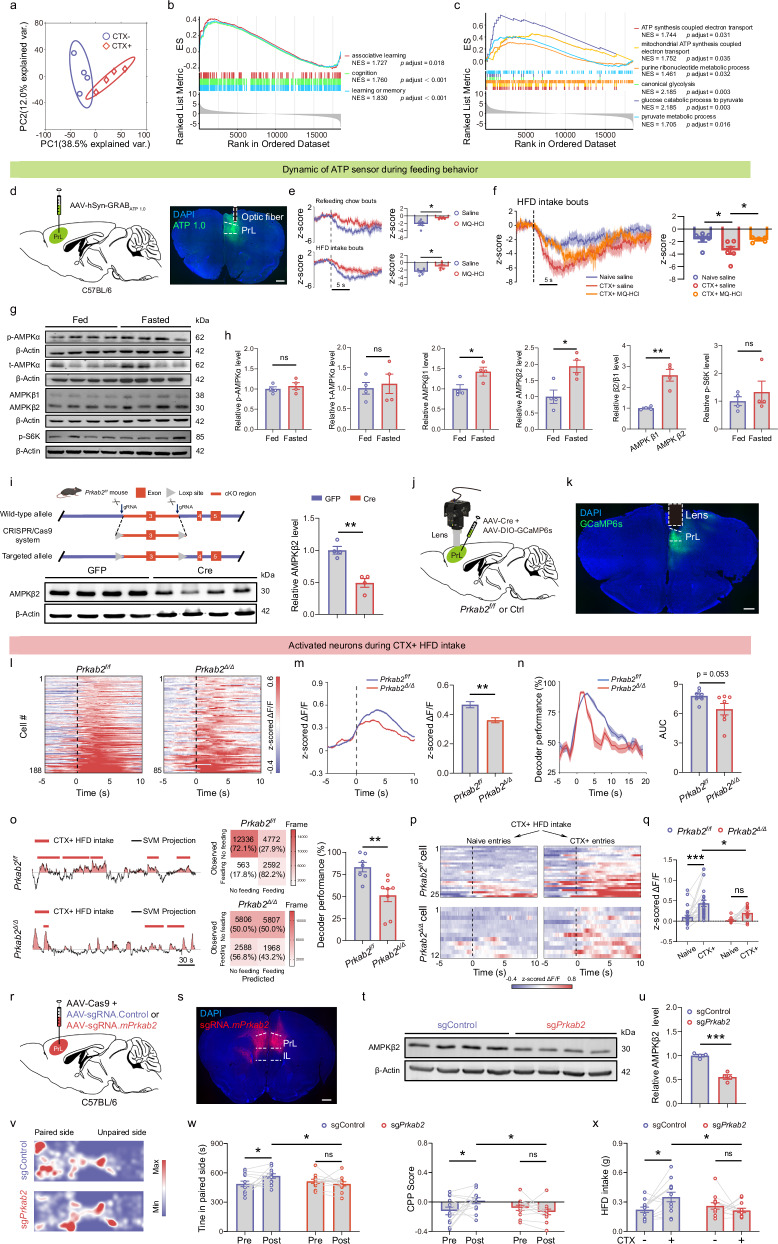
AMPKβ2 signaling in the PrL influences cue-potentiated HFD overconsumption. **a** Principal component analysis of gene expression in the PrL from mice re-exposed to CTX+ or CTX– contexts. **b,** **c** GSEA plots showing significantly enriched gene signatures in the PrL of CTX+ mice. **d** Left: Schematic of virus injection used to record ATP dynamics in the PrL. Right: Representative image showing the expression of the ATP fluorescent sensor and optic fiber location in the PrL. Scale bar, 500 μm. **e** z‑scored responses of the ATP sensor during chow refeeding and HFD intake in mice injected with saline or MQ‑HCl in the home cage. *n* = 6 (saline) and *n* = 5 (MQ‑HCl); unpaired two‑tailed *t*‑tests: refeeding, *P* = 0.011; HFD intake, *P* = 0.026. **f** Mean z‑scored responses (0–5 s) of the ATP sensor during HFD intake under Naive‑saline, CTX⁺‑saline, and CTX⁺‑MQ‑HCl conditions. *n* = 6 mice (naive and CTX⁺‑saline), and *n* = 5 mice (CTX⁺‑MQ‑HCl); one‑way ANOVA with Tukey’s multiple‑comparisons post hoc test: Naive‑saline vs CTX⁺‑saline, *P* = 0.028; CTX⁺‑saline vs CTX⁺‑MQ‑HCl, *P* = 0.045. **g** Immunoblot images showing AMPKα, AMPKβ, and p-S6K levels in the PrL of sated and fasted mice. **h** Quantification of p-AMPKα, t-AMPKα, AMPKβ1, AMPKβ2, and p-S6K levels in the PrL of sated and fasted mice. Unpaired *t*-test, **P < 0.01, *P < 0.05, ns not significant; *n* = 4 per condition. **i** Left: schematic for the generation of the *Prkab2*^*fl/fl*^ mouse line and immunoblot images of AMPKβ2 in the PrL following the injection of AAV expressing Cre or GFP. Right: expression level of AMPKβ2 in the PrL of *Prkab2*^*fl/fl*^ mice was decreased following the injection of AAV expressing Cre. Unpaired *t*-test, *n* = 4, **P < 0.01. **j** Schematic of miniscope imaging of PrL neurons in control and *Prkab2*^*fl/fl*^ mice. **k** Representative image showing the expression of GCaMP6s and the location of the GRIN lens in the PrL. Scale bar, 500 μm. **l** Heatmap of the neural activity of activated neurons during HFD consumption in paired chambers in *Prkab2*^*fl/fl*^ and *Prkab2*^Δ/Δ^ mice. *Prkab2*^*fl/fl*^ mice, 188 activated neurons out of 1938 total neurons in 7 mice; *Prkab2*^Δ/Δ^ mice, 85 activated neurons out of 1458 total neurons in 8 mice. **m** Average response of activated cells during HFD consumption in the paired chamber for *Prkab2*^*fl/fl*^ and *Prkab2*^Δ/Δ^ mice. Bar graphs show the mean z-scored ΔF/F of activated cells during the first 0–5 s of the feeding session. Mann–Whitney test, *P* = 0.0065. **n** Consecutive SVM decoder accuracy relative to behavior onset when discriminating between paired context and home cage during HFD feeding in *Prkab2*^*fl/fl*^ and *Prkab2*^Δ/Δ^ mice. The AUC of the decoder performance over 0–10 s was computed. Time 0 is aligned with the onset of HFD intake. Unpaired *t*-test, *P* = 0.053; 7 *Prkab2*^*fl/fl*^ and 7 *Prkab2*^Δ/Δ^ mice. **o** Left: decoder prediction of behavior using the population activity of neurons activated during CTX + HFD intake in *Prkab2*^*fl/fl*^ and *Prkab2*^Δ/Δ^ mice. The plots show the projections of population activity onto the SVM hyperplane, and the light patches show the decoder predictions. Scale bar, 30 s. Right: Comparison of the matrix between predicted events by activated neurons during CTX + HFD intake and the corresponding actual observed events in *Prkab2*^*fl/fl*^ and *Prkab2*^Δ/Δ^ mice. Bar graphs show the mean decoder performance during CTX + HFD intake bouts between *Prkab2*^*fl/fl*^ and *Prkab2*^Δ/Δ^ mice. Unpaired *t*-test, *P* = 0.004; 7 *Prkab2*^*fl/fl*^ and 8 *Prkab2*^Δ/Δ^ mice. **p** Heatmap showing the neuronal activity of the same CTX + HFD intake neurons upon entry into the paired chamber before (naive) and after (CTX+ ) conditioning training in *Prkab2*^*fl/fl*^ and *Prkab2*^Δ/Δ^ mice (25 cells for *Prkab2*^*fl/fl*^ mice and 12 cells for *Prkab2*^Δ/Δ^ mice). The dark dashed line is aligned with entry onset. Scale bars, 5 s. **q** Mean z-scored response (5–10 s) of CTX + HFD intake neurons upon entry into the paired chamber before (naive) and after (CTX+ ) conditioning training in *Prkab2*^*fl/fl*^ and *Prkab2*^Δ/Δ^ mice. Repeated-measures two-way ANOVA followed by Sidak’s multiple comparison post hoc test. F_(1, 35)_ = 5.137, *P* = 0.0297 for mice. *P < 0.05, ***P < 0.001, ns not significant; 25 cells for *Prkab2*^*fl/fl*^ mice and 12 cells for *Prkab2*^Δ/Δ^ mice. **r** Knockout of AMPKβ2 in the PrL using an AAV-mediated CRISPR-Cas9 strategy. **s** Expression of AAV encoding gRNA.mPrkab2 in the PrL. Scale bars, 500 μm. **t** Immunoblot images showing AMPKβ2 levels in the PrL following the injection of AAV expressing sgControl or sgPrkab2. **u** Expression level of AMPKβ2 was decreased in the PrL following the injection of AAV expressing sgPrkab2. Unpaired *t*-test, *n* = 4 per group, ***P < 0.001. **v** Heatmap representing the position of mice i*n* the experimental arena during the CPP test for sgControl and sgPrkab2 mice. **w** Time spent in the paired chambers and CPP scores for sgControl and sgPrkab2 mice. Two-way ANOVA followed by Sidak’s multiple comparison post hoc test. Time: F_(1, 44)_ = 5.208, *P* = 0.0274 for viruses and training; CPP scores: F_(1, 44)_ = 5.418, *P* = 0.0246 for viruses and training. *P < 0.05; ns not significant; *n* = 12 mice per group. **x** HFD intake in the CTX+ and CTX– chambers by sgControl and sgPrkab2 mice. Two-way ANOVA followed by Sidak’s multiple comparison post hoc test. F_(1, 44)_ = 6.605, *P* = 0.0136 for viruses and contexts. *P < 0.05; ns not significant; *n* = 12 mice per group.

Next, we sought to investigate the molecular mechanisms through which environmental contexts promote HFD overconsumption. We analyzed gene expression using RNA-Seq in the PrL regions of mice re-exposed to the HFD-paired (CTX+ group) and neutral (CTX– group) contexts. Principal component analysis (PCA) of gene expression revealed that CTX+ and CTX– mice formed separate groups, indicating that the major changes in gene expression patterns were dependent on conditioning learning (Fig. 4a). Further gene set enrichment analysis (GSEA) revealed that transcripts from CTX+ mice were positively enriched in gene signatures related to associative learning, cognition, learning or memory (Fig. 4b). Interestingly, we found that gene sets associated with ATP metabolic pathways were significantly upregulated in CTX+ mice (Fig. 4c), suggesting that ATP dynamics in the PrL may play a role in the conditioning learning of paired environmental contexts. Furthermore, under a nutrition‑deficit state, we observed that multiple metabolic pathways related to ATP metabolism, such as cellular response to starvation and glucose and pyruvate metabolic processes, were significantly upregulated in the PrL of fasted mice (Supplementary Fig. S4a, b). We then injected an AAV expressing a GPCR activation-based ATP sensor GRAB_ATP1.0_^54^ to monitor ATP dynamics in the PrL during feeding behavior (Fig. 4d). Surprisingly, we found that the ATP concentration was inversely related to both the duration of chow refeeding and the duration of HFD consumption (Fig. 4e). Furthermore, there was a greater reduction in ATP concentration during HFD intake in the conditioned context than in the neutral context (Fig. 4f). Additionally, ATP signals sharply decreased immediately prior to entry into the HFD-paired chamber, whereas no significant changes were observed prior to entry into the unpaired chamber (Supplementary Fig. S4c). Although the GRAB_ATP1.0_ sensor does not directly monitor intracellular ATP levels, extracellular ATP concentrations reflect a dynamic equilibrium influenced by intracellular energetic states, ATP efflux via connexin/pannexin hemichannels, and extracellular ATP hydrolysis^55–57^. To further investigate this mechanism, we administered mefloquine hydrochloride (MQ-HCl), an inhibitor of intracellular-to-extracellular ATP transport^58,59^. MQ-HCl significantly attenuated the reduction in ATP sensor signals observed during chow refeeding and HFD consumption bouts (Fig. 4e), as well as during HFD consumption in the CTX+ context (Fig. 4f). These results suggest that the observed reduction in ATP sensor signals is, at least partially, attributable to a decrease in intracellular ATP concentrations during feeding behaviors. Moreover, we analyzed and plotted the temporal dynamics of calcium and ATP signals during CTX + HFD intake bouts (Supplementary Fig. S4d). We found that neuronal Ca²⁺ signals increased prior to feeding-bout onset (approximately −0.5 s) whereas extracellular ATP signals decreased after bout onset (approximately +0.86 s). These results demonstrate that the increase in PrL neuronal activity precedes the decrease in ATP levels.

AMPK senses AMP-to-ATP and ADP-to-ATP ratios and acts as a master regulator of energy homeostasis^39–43^. AMPK signaling activation in the hypothalamus has been shown to enhance appetite and promote food intake^40,50,51^. Moreover, our RNA-Seq data revealed that the expression of the gene encoding the AMPKβ2 subunit, *Prkab2*, was significantly upregulated in the PrL of fasted mice (Supplementary Fig. S4e). As such, we investigated whether AMPK signaling in the prefrontal cortex is involved in regulating feeding behavior. Consistent with the findings of previous studies^50,60,61^, we found that fasting significantly increased the phosphorylation of the AMPKα subunit at threonine 172, a residue whose phosphorylation enhances AMPK activity^62^, in the hypothalamus (Supplementary Fig. S4f). In contrast, the expression levels of AMPKβ1 and AMPKβ2 remained unchanged in the hypothalamus (Supplementary Fig. S4g). Interestingly, fasting significantly increased the expression of AMPKβ1 and AMPKβ2 in the PrL, whereas the total levels and phosphorylation of AMPKα at Thr172, as well as the phosphorylation of S6K at Thr389, remained unchanged (Fig. 4g, h). These findings suggest that the AMPKβ subunit plays an essential role in the response of the PrL to nutritional deficiency. Given that the expression levels of AMPKβ2 were significantly higher than those of the β1 subunit in both the PFC and hypothalamus and that the increase in AMPKβ2 expression was more pronounced than that in AMPKβ1 expression in fasted mice (Fig. 4g, h; Supplementary Fig. S4g), we investigated the role of AMPKβ2 in feeding behavior in the PrL. We generated a *Prkab2*^flox/flox^ transgenic mouse line by inserting loxP sequences into introns 2 and 3 of the *Prkab2* gene (Fig. 4i). Upon Cre recombinase-mediated recombination, exon 3 was deleted from the *Prkab2* gene, resulting in a frameshift mutation. After an AAV expressing Cre recombinase was injected into the PrL, the expression level of AMPKβ2 was significantly lower than that in the control group injected with an AAV expressing GFP (Fig. 4i). The role of the AMPKβ2 subunit in the central nervous system remains largely unexplored. We used miniscope calcium imaging to assess whether AMPKβ2 depletion affects the neural dynamics of PrL neurons during feeding (Fig. 4j, k). Compared with the control group (*Prkab2*^fl/fl^), in the PrL group (*Prkab2*^Δ/Δ^), AMPKβ2 depletion significantly reduced both the mean and maximal neural activity in response to HFD feeding in the home cage (Supplementary Fig. S4h–j). These findings suggest that AMPKβ2 is crucial for the activation of PrL neurons in response to palatable food. Following HFD training paired with contextual chambers, *Prkab2* depletion in the PrL significantly diminished neural activity upon entry into the HFD-paired chamber, whereas no difference in neural activity was detected upon unpaired chamber entry (Supplementary Fig. S4k–p). These findings suggest that AMPKβ2 signaling in the PrL may play a role in discriminating environmental contexts associated with an HFD. We trained linear decoders using PrL population activity from neural ensembles activated upon entering the paired and unpaired contexts. In the control *Prkab2*^fl/fl^ mice, PrL population activity differentiated between paired and unpaired contexts, with peak performance occurring ~4 s after the mice entered the chambers (Supplementary Fig. S4m). However, depleting *Prkab2* in PrL neurons significantly reduced decoder accuracy. Notably, the decoder performance decreased to 49% within 5 s, in contrast to the control, which maintained over 77% accuracy at the same interval (Supplementary Fig. S4m). Thus, our data demonstrate that AMPKβ2 signaling is required for the discrimination of environmental contexts associated with palatable food. Moreover, by analyzing the neural dynamics of PrL neurons during HFD consumption in paired contexts, we observed that *Prkab2*^Δ/Δ^ reduced neuronal activity during HFD feeding bouts in these contexts (Fig. 4l, m). Furthermore, linear decoders trained on the population activity of ensembles activated during HFD-feeding bouts showed reduced decoder performance in *Prkab2*^Δ/Δ^ mice (Fig. 4n), demonstrating the crucial role of AMPKβ2 signaling in the PrL for accurately differentiating between conditioned contexts during HFD feeding.

In wild-type (WT) mice, we observed that PrL neurons exhibited activity-dependent plasticity and that the activity of these neurons predicted the duration of HFD consumption in conditioned environments. We then investigated whether AMPKβ2 depletion from the PrL affects this plasticity and, consequently, alters the decoding pattern of these neurons in response to an HFD in conditioned environments. We observed that the depletion of *Prkab2* significantly impaired the ability of PrL neurons to predict HFD feeding events in the conditioned chamber (Fig. 4o). Moreover, the depletion of Prkab2 substantially reduced the activity-dependent plasticity of CTX + HFD intake neurons during conditioned training to pair environmental contexts with palatable food (Fig. 4p, q). In WT mice, we observed substantial convergence in the neuronal encoding of environmental contexts and HFD consumption, which may contribute to the appetitive drive triggered by contextual cues (Supplementary Fig. S2i–l). Further analysis of the impact of AMPKβ2 signaling on shared neural activity in response to environmental contexts and HFD consumption revealed that *Prkab2*^Δ/Δ^ significantly reduced both the number and average responses of CTX+ neurons activated before HFD feeding bouts (pre-cells) and during the consummatory phase (post-cells, Supplementary Fig. S4q–s).

We subsequently investigated whether PrL AMPKβ2 regulates cue-potentiated palatable food consumption. We selectively deleted *Prkab2* in the PrL of *Prkab2*^fl/fl^ mice by injecting an AAV expressing Cre (control mice received an AAV expressing GFP; Supplementary Fig. S5a, b). Following paired-context training with an HFD, control mice exhibited a conditioned place preference for the HFD-paired context, whereas Cre-injected knockout mice failed to develop conditioned place preference (CPP) (Supplementary Fig. S5c, d). Furthermore, the control mice consumed significantly more of the HFD in the paired context (CTX+ ) than in the unpaired context (CTX–), whereas the knockout mice showed no difference between contexts (Supplementary Fig. S5e). These results indicate that PrL AMPKβ2 is required for cue-potentiated HFD consumption. We then assessed whether this effect generalized to another palatable food. Following contextual training with a high-sucrose diet (HSD), control mice again developed CPP in the HSD-paired context, whereas *Prkab2* knockout in the PrL abolished this effect (Supplementary Fig. S5f). Importantly, PrL-specific *Prkab2* deletion did not impair general cognitive functions, as performance in novel object recognition and contextual fear conditioning tasks was comparable between groups (Supplementary Fig. S5g, h).

Additionally, we employed an alternative strategy involving the use of CRISPR/Cas9 technology to knock out AMPKβ2 in the PrL (Fig. 4r–u). Similarly, deletion of *Prkab2* in the PrL impaired CPP in HFD-paired contexts and prevented cue-potentiated HFD overconsumption (Fig. 4v–x). Furthermore, during prolonged HFD exposure, mice lacking PrL AMPKβ2 exhibited attenuated body weight gain (Supplementary Fig. S5i, j). Collectively, our results demonstrate that AMPKβ2 signaling in PrL neurons is essential for the establishment of conditioned place preference for contexts associated with palatable food and critically regulates HFD overconsumption.

### Activity of the PrL→LH^orexin+^ circuit regulates HFD intake in associated contexts

**Fig. 5:**
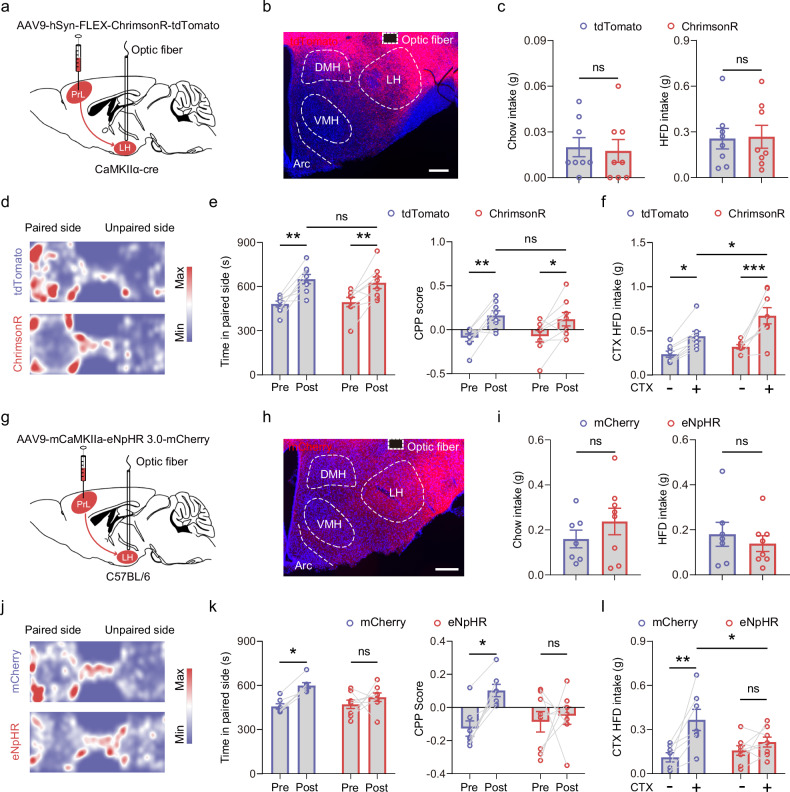
Activity of the PrL → LH circuit mediates place preference and elicits HFD overconsumption in paired contexts. **a** Diagram of virus injection and the optogenetic activation of the PrL→LH circuit. **b** Representative image showing the distribution of PrL^CaMKIIα+^ neuronal axon terminals in the hypothalamus and the location of the optic fiber in the LH. Scale bar, 200 μm. **c** Chow and HFD intake measured after the optogenetic stimulation of mice expressing tdTomato or ChrimsonR. Mann–Whitney test, ns not significant, *n* = 8 mice per group. **d** Heatmaps representing the position of mice in the experimental arena during the CPP test with the optogenetic activation of the PrL→LH neuronal circuit. **e** Time spent in the paired chambers and CPP scores with the closed-loop stimulation of the PrL→LH neuronal circuit in mice expressing tdTomato or ChrimsonR. Repeated-measures two-way ANOVA followed by Sidak’s multiple comparison post hoc test. F_(1, 14)_ = 30.52, *P* < 0.0001 for time; F_(1, 14)_ = 19.37, *P* = 0.0006 for CPP scores. *P < 0.05, **P < 0.01, ns not significant; *n* = 8 mice per group. **f** HFD intake in the CTX+ and CTX– chambers measured in mice expressing tdTomato or ChrimsonR in the PrL, with the optogenetic stimulation of the PrL→LH neuronal circuit. Repeated-measures two-way ANOVA followed by Sidak’s multiple comparison post hoc test. F_(1, 14)_ = 40.01, *P* < 0.0001 for contexts. *P < 0.05, **P < 0.01, and ***P < 0.001. *n* = 8 mice per group. **g** Diagram of virus injection and the optogenetic inhibition of the PrL→LH neuronal circuit. **h** Representative image showing the distribution of eNpHR-expressing axon terminals in the hypothalamus and the location of the optic fiber in the LH. Scale bar, 200 μm. **i** Measurement of chow intake in fasted mice and HFD intake in sated mice with the optogenetic inhibition of the PrL→LH neuronal circuit. mCherry, 7 mice; eNpHR, 8 mice. ns not significant. **j** Heatmaps representing the position of mice in the experimental arena during the CPP test with the optogenetic inhibition of the PrL→LH neuronal circuit. **k** Time spent in the paired chambers and CPP scores for mice expressing mCherry or eNpHR with the optogenetic inhibition of the PrL→LH neuronal circuit. Repeated-measures two-way ANOVA followed by Sidak’s multiple comparison post hoc test. F_(1, 13)_ = 10.23, *P* = 0.007 for time; F_(1, 13)_ = 5.082, *P* = 0.0421 for CPP scores. *P < 0.05; ns not significant. mCherry, 7 mice; eNpHR, 8 mice. **l** HFD intake in the CTX+ and CTX– chambers under the optogenetic inhibition of the PrL→LH neuronal circuit. Repeated-measures two-way ANOVA followed by Sidak’s multiple comparison post hoc test. F_(1, 13)_ = 13.86, *P* = 0.0026 for contexts. *P < 0.05 and **P < 0.01 indicate statistical significance; ns not significant. mCherry, 7 mice; eNpHR, 8 mice.

Next, we sought to investigate the circuit mechanisms underlying how PrL^CaMKIIα+^ neurons mediate HFD overconsumption elicited by environmental contexts. The hypothalamus is an integrative center that coordinates internal energy status with external cues to regulate the homeostatic and hedonic control of feeding behavior^1,63–65^. We hypothesized that the PrL mediates excessive HFD intake through its connections with hypothalamic areas. We injected an AAV expressing EGFP into the PrL and traced the distribution of its axonal projections in hypothalamic areas (Supplementary Fig. S6a, b). We found that projections from the PrL to the LH were notably dense, projections from the PrL to the dorsomedial hypothalamic nucleus (DMH) and the arcuate nucleus (Arc) were relatively sparse, and projections from the PrL to the paraventricular hypothalamic nucleus (PVH) and the ventromedial hypothalamic nucleus (VMH) were negligible (Supplementary Fig. S6c). The LH serves as a central hub, integrating hormonal signals and neural inputs from various brain regions to regulate feeding behavior^66,67^. Notably, compared with chow consumption, HFD consumption significantly increased the number of Fos^+^ cells in the LH (Supplementary Fig. S6d). Furthermore, compared with re-exposure to the HFD-unpaired chamber, upon re-exposure to the HFD-paired chamber, mice had significantly higher numbers of Fos^+^ cells in the LH (Supplementary Fig. S6e). Thus, we investigated whether PrL→LH circuit activity is required for HFD overconsumption elicited by environmental contexts. We injected an AAV encoding ChrimsonR into the PrL and implanted optical fibers into the LH, enabling us to specifically activate the PrL→LH circuit (Fig. 5a, b). Similar to activating the somata of PrL^CaMKIIα+^ neurons, the light stimulation of ChrimsonR-expressing axon terminals in the LH did not affect the intake of either chow or HFD by unconditioned mice (Fig. 5c). However, compared with the stimulation of tdTomato-expressing axon terminals, optogenetic stimulation significantly increased HFD intake in the paired chamber (Fig. 5f). Increased activity in the PrL→LH neural circuit in paired contexts promoted HFD intake, which was unlikely due to the stimulation altering the preference for the HFD-paired chamber, as both groups of mice spent similar amounts of time in the chambers and had comparable CPP scores (Fig. 5d, e). Conversely, inhibiting the PrL→LH circuit through the bilateral light stimulation of axon terminals in the LH that expressed the inhibitory opsin eNpHR (Fig. 5g, h) did not affect chow or HFD intake by unconditioned mice (Fig. 5i), but eliminated place preference for the paired chamber and prevented HFD overconsumption (Fig. 5j–l). Together, these results demonstrate that the activity of the PrL→LH neural circuit is essential for establishing a preference for HFD-associated contexts and for eliciting HFD overconsumption in response to environmental contexts.

**Fig. 6:**
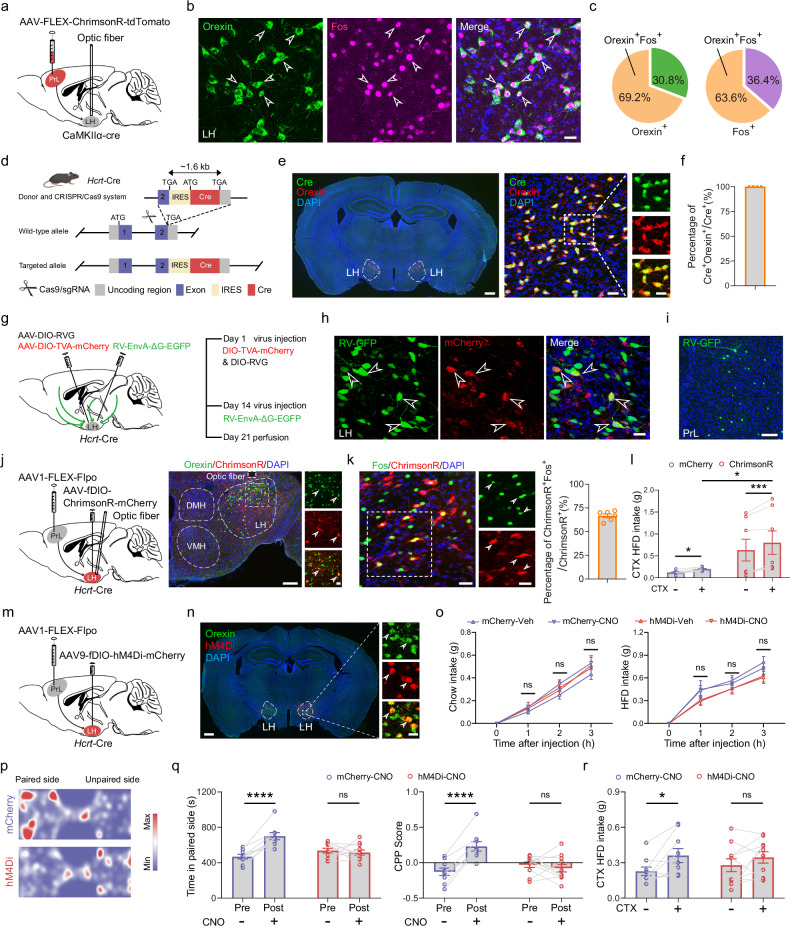
Activity of the PrL→LH^orexin+^ circuit regulates HFD intake in associated contexts. **a** Schematic of virus injection and the optogenetic activation of the PrL→LH pathway. **b** Representative images showing the colocalization of Fos^+^ and Orexin^+^ neurons in the LH following the stimulation of the PrL-LH pathway. Scale bar, 25 μm. **c** Percentage of Orexin^+^Fos^+^ cells among total Orexin^+^ cells and among total Fos^+^ cells. *n* = 5 mice. **d** Schematic of *Hcrt*-cre knock-in mouse generation via CRISPR-Cas9. **e** Representative images showing Orexin and Cre staining in the *Hcrt*-cre transgenic mouse line. Scale bars, 500 μm (left panel) and 30 μm (middle and right panels). **f** Percentage of Cre^+^Orexin^+^ neurons among Cre^+^ neurons. **g** Diagram illustrating rabies virus-based monosynaptic retrograde tracing of inputs to orexin neurons. **h** Starter cells (yellow) in the LH. Green fluorescence signals for RV-EnvA-ΔG-infected neurons and red fluorescence signals for helper virus-infected neurons. Scale bar, 20 μm. **i** Labeling of monosynaptic inputs from the PrL to LH^orexin+^ neurons. Scale bar, 100 μm. **j** Left: Strategy for the optogenetic stimulation of the PrL→LH^Orexin+^ circuit. Right: Representative images showing orexin neurons (green) colocalized with transsynaptically labeled neurons from the PrL. Scale bars, 200 μm (left) and 30 μm (right). **k** Representative images showing the colocalization of Fos⁺ cells with ChrimsonR‑labeled PrL‑projecting orexin neurons in the LH after context‑paired HFD feeding. Scale bar, 30 μm. *n* = 6 mice. **l** HFD intake in CTX+ and CTX– chambers under the optogenetic stimulation of the PrL→LH^Orexin+^ circuit (tdTomato vs ChrimsonR). Repeated-measures two-way ANOVA followed by Sidak’s multiple comparison post hoc test. F_(1, 13)_ = 5.366, *P* = 0.037 for the virus. *P < 0.05, ***P < 0.001. mCherry, 8 mice; ChrimsonR, 7 mice. **m** Diagram illustrating the virus injection strategy for the chemogenetic inhibition of the PrL→LH^Orexin+^ circuit. **n** Representative images showing orexin neurons in the LH colocalized with transsynaptically labeled neurons from the PrL. Scale bars, 500 μm (left) and 30 μm (right). **o** Chow and HFD intake following the chemogenetic inhibition of the PrL→LH^Orexin+^ pathway. mCherry, 9 mice; hM4Di, 10 mice. ns not significant. **p** Heatmaps representing the position of mice in the experimental arena during the CPP test with the chemogenetic inhibition of the PrL→LH^Orexin+^ pathway. **q** Time spent in the paired chambers and CPP scores following chemogenetic inhibition of the PrL→LH^Orexin+^ pathway. Two-way ANOVA followed by Sidak’s multiple comparison post hoc test. F_(1, 36)_ = 11.98, *P* = 0.0014 for time; F_(1, 36)_ = 8.793, *P* = 0.0053 for CPP scores. ****P < 0.0001; ns not significant. mCherry, 9 mice; hM4Di, 11 mice. **r** HFD intake in the CTX+ and CTX– chambers measured in mice under the chemogenetic inhibition of the PrL→LH^Orexin+^ pathway. Repeated-measures two-way ANOVA followed by Sidak’s multiple comparison post hoc test. F_(1, 17)_ = 7.423, *P* = 0.0144 for contexts. *P < 0.05; ns not significant. mCherry, 9 mice; hM4Di, 10 mice.

We further investigated the cellular mechanisms through which the PrL→LH circuit regulates cue-potentiated HFD overconsumption. Several neuronal populations in the LH play important roles in regulating feeding, including orexin neurons, melanin-concentrating hormone (MCH) neurons, and GABAergic neurons^66,67^. We initially employed optogenetics to stimulate the ChrimsonR-expressing axon terminals of the PrL projecting to the LH. Subsequent immunofluorescence staining revealed that 63.6% of the Fos^+^ cells were orexin positive, whereas only 17% were GABA positive and 5% were MCH positive (Fig. 6a–c; Supplementary Fig. S7a–d). These results suggest that the activation of the PrL→LH circuit predominantly activates orexin neurons in the LH. Using CRISPR/Cas9 technology, we generated an *Hcrt*-cre transgenic mouse line by inserting an internal ribosome entry site (IRES) and a Cre recombinase gene (cre) sequence immediately downstream of the endogenous stop codon at the hypocretin (Hcrt) locus on chromosome 11 (Fig. 6d). The transgenic mouse line was confirmed through double immunostaining using specific antibodies against Cre and endogenous orexin. We found that Cre recombinase expression was exclusive to orexin-positive neurons, indicating that the *Hcrt*-cre mouse accurately replicates the endogenous expression pattern of orexin (Fig. 6e, f). To confirm the monosynaptic input from the PrL into LH^orexin+^ neurons, we injected two Cre-dependent helper viruses, AAV-DIO-TVA-mCherry and AAV-DIO-RG, into the LH (Fig. 6g). These viruses provided the orexin neurons with the receptor for EnvA-coated RV entry and the glycoprotein required for RV transmission to monosynaptic input neurons. Two weeks later, RV-EnvA-ΔG-EGFP was injected into the same site (Fig. 6g). After seven days, we observed a significant number of dual-color-labeled starter neurons at the injection site (Fig. 6h). Importantly, a substantial number of PrL neurons were labeled by the RV (Fig. 6i), demonstrating monosynaptic input from the PrL to LH^orexin+^ neurons.

Next, we aimed to determine whether the PrL→LH^orexin+^ circuit is essential for environmental contexts to elicit HFD overconsumption. To specifically target LH^orexin+^ neurons that receive projections from PrL neurons, we took advantage of the anterograde transsynaptic properties of AAV1 to transduce from the injection site to specific postsynaptic neurons^68^. In the *Rosa26*^Ai14/+^ reporter mouse line, we injected AAV1 expressing Cre recombinase into the PrL and found a considerable number of tdTomato-expressing cells in the LH (Supplementary Fig. S7e, f), indicating that Cre recombinase-expressing AAVs were anterogradely transported from the PrL injection site to the LH. Moreover, a significant proportion of these tdTomato-positive cells were also orexin positive (Supplementary Fig. S7g), demonstrating that this strategy effectively targeted the PrL→LH^orexin+^ circuit.

We next injected an AAV1 virus expressing Flpo recombinase in a Cre-dependent manner, along with an AAV expressing ChrimsonR, into the LH of *Hcrt*-cre mice, enabling the selective activation of orexin neurons receiving projections from PrL neurons (Fig. 6j). Following paired-context HFD feeding, ~66.7% of LH^orexin+^ neurons receiving PrL inputs were activated (Fig. 6k). Strikingly, compared with control treatment, the optogenetic activation of PrL-projecting orexin neurons significantly increased HFD intake in the paired context (Fig. 6l). We then employed a chemogenetic approach to further examine whether inhibiting the PrL→LH^orexin+^ circuit could mitigate cue-potentiated HFD overconsumption. We simultaneously injected an AAV virus into the LH to express Gi/o-coupled designer receptors exclusively activated by a designer drug (hM4Di-DREADD)^69,70^ (Fig. 6m, n). The chemogenetic inhibition of PrL→LH^orexin+^ neurons did not affect chow or HFD intake in the home cage (Fig. 6o). However, this inhibition significantly impacted the preference of mice for HFD-paired contexts after paired training, as well as the overconsumption of the HFD elicited by environmental contexts (Fig. 6p–r). Interestingly, inhibiting the activity of PrL→LH^orexin+^ neurons did not affect the retrieval of contextual fear memory (Supplementary Fig. S7h, i), suggesting that these neurons selectively regulate contextual place preferences related to food cues rather than broadly affecting the retrieval of spatial memory. Overall, these findings indicate that orexin neurons receive monosynaptic inputs from the PrL and play an essential role in cue-potentiated HFD overconsumption.

## Discussion

In this study, we demonstrate that PrL neurons exhibit activity-dependent plasticity in response to associative learning with environmental contexts paired with palatable food. Although our optogenetic manipulations (activation or inhibition of PrL^CaMKIIα⁺^ neurons) did not exclusively target neuronal ensembles naturally recruited during cue-potentiated feeding behavior, our findings nonetheless demonstrate that the activity of PrL^CaMKIIα⁺^ neurons is both necessary and sufficient for establishing conditioned place preference and bidirectionally regulating context-driven HFD overconsumption. This phenomenon likely occurs because sensory cues from environmental contexts are integrated by the PrL, priming appetite-responsive neurons and potentially enhancing appetitive drive and food consumption. Our miniscope calcium imaging data provided evidence of substantial shared activation in the neuronal encoding of environmental cues and HFD consumption. Notably, neurons responsive to HFD-paired contexts activate before HFD consumption, suggesting that appetitive drive mechanisms may be triggered by contextual cues. In today’s obesogenic environment, where palatable food cues are pervasive, accumulating evidence suggests that obesity may be related to increased motivation elicited by environmental cues that promote palatable food intake in humans and rodents^71–74^. Our findings reveal that prefrontal cortical neurons exhibit activity-dependent plasticity, altering their response to environmental contexts and influencing appetite. These findings suggest that interventions targeting prefrontal cortex activity could be effective strategies for treating excessive appetitive drive and food consumption.

We observed robust projections from the PrL to LH^orexin+^ neurons, and this circuit was essential for cue-potentiated conditioned place preference and HFD overconsumption. The LH serves as a hub, integrating hormonal signals and neural inputs from other brain regions, and plays a central role in mediating feeding motivation and food consumption^66,67^. Early pioneering studies reported that the electrical stimulation of the LH promoted the learning of food-reinforced instrumental responses and exerted motivational effects that were functionally equivalent to those of food deprivation^75,76^. At the cellular level, orexinergic neurons are considered to translate motivational activation into organized responses that support adaptive behaviors. This function is an integrated response to motivational relevance, often arising in the context of physiological needs such as during food scarcity, threats, or reward opportunities^77–79^. Thus, it is plausible that sensory information perceived from environmental contexts is integrated by the PrL and conveyed to LH^orexin+^ neurons, thereby facilitating the motivational drive toward an HFD and initiating HFD overeating through extensive connections with the brainstem and other hypothalamic areas^66^. This circuit may represent an evolutionarily conserved mechanism that associates environmental cues with palatable, energy-dense foods through associative learning. This is a crucial adaptation mechanism, particularly in resource-scarce environments, as it motivates the search for and consumption of nutrient-rich foods. However, in today’s obesogenic environment, where high-calorie foods are readily available, this circuit may inadvertently contribute to the excessive consumption of highly palatable foods beyond metabolic needs, exacerbating the obesity crisis^23,25^. Nevertheless, our findings reveal that the interconnected neural pathway of the PrL^CaMKIIα+^→LH^orexin+^ circuit is crucial for the integration of environmental cues to elicit appetitive responses and facilitate food consumption, providing insights into the top-down regulation of excessive eating.

We demonstrated the importance of the PrL→LH circuit in mediating cue-potentiated HFD consumption. Interestingly, the optogenetic stimulation of PrL^CamKIIa+^ neuronal axon terminals in the LH did not alter the amount of food intake in unconditioned mice. This finding aligns with a previous study showing that the optogenetic stimulation of the mPFC→LH circuit did not affect food intake in the home cage^80^. In contrast, another study reported that the mPFC→LH circuit plays a significant role in stress-sensitive anorexigenic responses and that the optogenetic activation of this circuit significantly suppresses food intake^36^. This apparent inconsistency could be explained by differences in the brain regions examined (i.e., the PrL vs the entire mPFC) and by differences in activation specificity, as in the study by Clarke et al. ^36^, a retrograde AAV was injected into the LH and activated the neuronal soma in the mPFC. This strategy, however, may not faithfully represent the function of the mPFC→LH circuit because it could activate mPFC projections to other brain regions, given that a single mPFC neuron has been found to send contralateral projections to multiple brain regions^81^. Indeed, a previous study revealed that in a go/no-go task, prefrontal cortex pyramidal neurons projecting to the LH showed strong and sustained excitation during ‘go’ trials, indicating a role in promoting motivation and action. Conversely, the PFC→subthalamic nucleus pathway has the opposite effect^82^. Furthermore, the LH harbors heterogeneous neuronal populations that regulate feeding behavior, including orexin^+^ neurons that promote feeding and vglut2^+^ neurons that suppress feeding in food-deprived mice^83,84^. Further studies are needed to investigate how the activity of neuronal populations other than orexin in the PrL → LH circuit responds to various environmental sensory cues, thereby influencing feeding behavior.

We found that AMPK activity plays a critical role in the cognitive regulation of feeding behavior. Interestingly, the regulation of AMPK activity displays brain region- and isoform-specific patterns during energy deficiency. In the hypothalamus, AMPK activity primarily increases through the phosphorylation of the α subunit at the Thr172 residue, whereas in the PrL, this regulation occurs through the modulation of the expression levels of the AMPKβ subunit. This indicates that the regulatory patterns and functions of AMPK vary across brain regions^39^. The functions of AMPKβ in the central nervous system are largely unknown, despite both the AMPKβ1 and AMPKβ2 subunits being abundantly expressed in the brain^85^. Consistent with recent findings that the conditional knockout of the AMPKβ2 isoform in CaMKIIα^+^ neurons impaired hippocampal synaptic plasticity^86^, our study demonstrated the indispensable role of AMPKβ2 in the PrL in facilitating activity-dependent plasticity during HFD associative learning. This plasticity is crucial for establishing place preferences to HFD-paired contexts and mediating the overconsumption of palatable food driven by environmental contexts, highlighting that β2-containing AMPK complexes play a vital role in the retrieval of contextual memories associated with an HFD. Indeed, a previous study revealed that AMPK activation is crucial for synaptic plasticity in AGRP neurons during food deprivation^87^, suggesting that AMPK activity not only regulates homeostatic feeding but also shapes appetite through cognitive regulation. However, the downstream molecular mechanisms through which AMPKβ2 regulates feeding behavior plasticity remain incompletely understood. Our analysis revealed that fasting-induced elevation in feeding motivation significantly increased AMPKβ2 expression in the PrL without altering the phosphorylation of AMPKα at Thr172 or S6K at Thr389, a effector downstream of the mTOR pathway. These results suggest that feeding behavior regulated by the PrL may involve pathways distinct from the AMPKα–mTOR–S6K axis described in other brain regions. Future studies are necessary to uncover additional downstream molecular effectors and elucidate mechanisms through which AMPKβ2 modulates neuronal function and feeding behavior in the mPFC.

A limitation of our study is the inability to directly measure intracellular ATP dynamics in neurons. While genetically encoded fluorescent probes currently allow the real-time monitoring of neuronal calcium and extracellular ATP dynamics at second-level temporal resolution, comparable probes suitable for reliably measuring intracellular ATP concentrations at these timescales in freely behaving animals have not yet been validated. Directly assessing the relationship between intracellular ATP levels and neuronal activity in vivo, particularly during context-dependent HFD intake, would provide clearer experimental evidence.

In summary, we demonstrate the circuitry and molecular mechanisms that mediate HFD overconsumption driven by environmental contexts. Because environmental cues that potentiate palatable food overeating are significant factors in the obesogenic environment, our findings present a novel neural pathway and molecular target for treating eating disorders.

## Materials and methods

### Animals

CaMKIIα-cre mice (strain 005359) were obtained from The Jackson Laboratory. The *Rosa26*^Ai14/+^ (Jackson Laboratory, strain 007914) mouse line was obtained from Dr. Miao He (Institutes of Brain Science, Fudan University). Customized transgenic mice, including *Hcrt*-IRES-Cre and *Prkab2*^*fl/fl*^ mice, were obtained from GemPharmatech and Cyagen, respectively. All experimental procedures were approved by the Animal Care and Use Committee of the Department of Laboratory Animal Science, Fudan University. In this study, both male and female C57BL/6 J and transgenic CaMKIIα-cre, *Rosa26*^Ai14/+^, *Hcrt*-cre and *Prkab2*^*fl/fl*^ mice were used. All CaMKIIα-cre and *Hcrt*-cre transgenic mice used in the study were heterozygous. All the mice used for stereotaxic surgery were at least 8 weeks old. Animals were maintained on a 12-h dark/light cycle and had ad libitum access to food and water, except during experiments involving fasted refeeding and context-paired feeding. Mouse behaviors were tested during the dark (active) phase of a 12 h/12 h reversed light-dark cycle. In all the experiments involving HFD intake, the mice were pre-exposed to an HFD for three consecutive days prior to testing. Although viral vector controls were included in our study, sham-operated and nonviral control mice were not used.

### Generation of *Hcrt*-cre and *Prkab2*^*fl/fl*^ mice

The *Hcrt*-cre mouse line was generated by targeting exon 2 of the *Hcrt* gene using the CRISPR/Cas9 system. Cas9 mRNA, sgRNA and a donor were co-injected into zygotes. The sgRNA directed the Cas9 endonuclease to cleave the stop codon in exon 2 of the *Hcrt* gene, creating a double-strand break. This break was repaired via homologous recombination, resulting in the insertion of IRES-Cre immediately after the stop codon of the *Hcrt* gene. For genotyping, a forward primer (5′-TGCCGTCTCTACGAACTGTTGCA-3′), a mutant reverse primer (5′-TAGACAAACGCACACCGGCCTT-3′), and a WT reverse primer (5′-CATTTCCACCCTCCCAAATGTGTA-3′) were used.

The *Prkab2*^*fl/fl*^ mouse line was generated by targeting exon 3 of the *Prkab2* gene to insert two loxP sites using the CRISPR/Cas9 system. Transgenic mice were genotyped using a forward primer (5′-TTTAGTAACGGATCTGCTTTTGCTC-3′) and a reverse primer (5′-ATCATCCATCAATCGACAGGCAT-3′).

### Immunofluorescence staining

Mice were deeply anesthetized with pentobarbital sodium (100 mg/kg, i.p.) and perfused with 1× PBS, followed by 4% paraformaldehyde (PFA) in PBS. Brain tissues were collected and fixed in 4% PFA for 6 h, immersed in a 30% sucrose solution for 48 h, and subsequently embedded in O.C.T. compound (Sakura) for cryosectioning. 40 μm-thick brain sections were collected using a cryostat (Leica, CM1950) and stored in an anti-freezing solution (25% ethylene glycol and 25% glycerol in PBS). For immunofluorescence staining, the brain sections were initially washed in 1× TBS for 5 min and then blocked with a solution containing 0.4% Triton X-100, 1% glycine, 1% bovine serum albumin, and 10% donkey serum in TBS for 2 h at room temperature. The sections were incubated overnight at 4 °C with primary antibodies. After being washed with TBS three times for 20 min each, the sections were incubated with a secondary antibody solution for 90 min at room temperature, followed by additional washing with TBS three times for 20 min each. Finally, the sections were counterstained with DAPI and mounted on slides for imaging. The following primary antibodies were used in this study: Guinea pig anti-c-Fos (Synaptic Systems, 226004, 1:10,000); mouse anti-CaMKIIα (Cell Signaling Technology, 50049S, 1:2000); rabbit anti-GFP (Elabscience, E-AB-40511, 1:2000); rabbit anti-orexin-A (Abcam, ab6214, 1:2000); guinea pig anti-Cre (Synaptic Systems, 257004, 1:4000); rabbit anti-MCH (Sigma, M8440, 1:2000); and rabbit anti-GABA (Sigma, A2052, 1:500). All secondary antibodies (donkey anti-guinea pig IgG Alexa 488, 706-545-148; donkey anti-mouse IgG Alexa 594, 715-585-150; donkey anti-rabbit IgG Alexa 488, 711-545-152; donkey anti-guinea pig IgG Alexa 647, 106-605-003; and donkey anti-rabbit IgG Alexa 594, 711-585-152) were purchased from Jackson ImmunoResearch and used at a dilution of 1:500. Images were acquired using a Nikon ECLIPSE Ti2 confocal microscope. In the PrL, Fos expression was quantified in sections corresponding to Bregma +1.78 mm to +1.98 mm. For the LH, quantification was performed on sections spanning Bregma –1.34 mm to –1.58 mm.

### Western blotting

Brain tissues were lysed on ice for 30 min in RIPA lysis buffer (Beyotime, P0013B) supplemented with freshly added protease inhibitors (Roche, 11873580001) and phosphatase inhibitors (Roche, 049068450001). The lysates were subsequently centrifuged at 12,000 rpm for 10 min at 4 °C, and the supernatants were collected as protein extracts. These extracts were subsequently resolved by SDS-PAGE and then transferred onto PVDF membranes. The membranes were blocked with 5% nonfat milk for 1 h at room temperature and then incubated overnight at 4 °C with primary antibodies. Afterward, the membranes were incubated with secondary antibodies for 1 h at room temperature. Following three washes in TBST, the membranes were incubated with an enhanced chemiluminescence (ECL) reagent (Beyotime, P0018AS) and imaged using an e-Blot Touch Imager system. The relative density of the protein bands was quantified using ImageJ software. The following primary antibodies were used in this study: mouse anti-AMPKα (F6) (Cell Signaling Technology, 2793S, 1:2000), rabbit anti-phospho-AMPKα (Thr172) (Cell Signaling Technology, 2535S, 1:2000), rabbit anti-AMPKβ1/2 (Cell Signaling Technology, 4150S, 1:2000), and rabbit anti-phospho-S6K (Thr389) (Cell Signaling Technology, 9205S, 1:1000).

### Viruses and reagents

AAV2/9-hEF1a-DIO-GCaMP6s-WPRE-pA (S0351-9), AAV2/9-mCaMKIIa-ChrimsonR-tdTomato-WPRE-pA (S0219-9), AAV2/9-mCaMKIIa-mCherry-WPRE-pA (S0242-9), AAV2/9-mCaMKIIa-eNpHR 3.0-mCherry-WPRE-pA (S0464-9), AAV2/9-hSyn-FLEX-ChrimsonR-tdTomato-WPRE-Pa (S0186-9), AAV2/9-hSyn-FLEX-tdTomato-WPRE-pA (S0255-9), AAV2/9-mCaMKIIa-EGFP-WPRE-pA (S0241-9), AAV2/5-hEF1a-DIO-RVG-WPRE-pA (S0325-5), AAV2/9-CAG-DIO-TVA-mCherry-WPRE-pA (S0683-9), AAV2/1-CAG-FLEX-Flpo-WPRE-pA (S0273-1), AAV2/9-hEF1a-fDIO-hM4D(Gi)-mCherry-ER2-WPRE-Pa (S0336-9), AAV2/9-hEF1a-fDIO-mCherry-WPRE-pA (S0553-9), AAV2/9-hEF1a-fDIO-ChrimsonR-mCherry-WPRE-pA (S0384-9), AAV2/9-hSyn-Cre-WPRE-pA (S0278-9), AAV2/9-hSyn-DIO-EGFP-WPRE-pA (S0746-9) and AAV1-hSyn-Cre-pA (S0292-1) were purchased from Taitool Bioscience. CRISPR-Cas9 viruses, including AAV2/9-mMecp2-spCas9 (S0195-9), AAV2/9-CAG-spCas9(WT)-WPRE-pA (S0381-9), AAV2/9-U6-sgRNA.sp(NC)-hSyn-mCherry-WPRE-pA and AAV2/9-U6-(sgRNA.sp(mPrkab2))×3-hSyn-mCherry-WPRE-pA, were constructed and produced by Taitool Bioscience. rAAV-hSyn-GRAB_ATP1.0_ was purchased from Braincase (BC-0288), and RV-EnvA-ΔG-EGFP (R01001) was purchased from BrainVTA. Clozapine-N-oxide (16882) and DAPI (D489987) were purchased from Cayman Chemical and Aladdin, respectively. A high-fat diet (D12492) was purchased from Research Diets, Inc.

### Stereotaxic injection of virus

Eight-week-old mice were anesthetized with an intraperitoneal (i.p.) injection of pentobarbital sodium (100 mg/kg) and positioned on a stereotaxic apparatus (68037, RWD). Recombinant AAVs were delivered into specific brain regions using a microsyringe pump controller (53311, Stoelting). Volumes of 200–400 nL were injected at a flow rate of 30 nL/min. The injection pipette was retained at the injection site for 10 min post-delivery to ensure adequate viral diffusion. The following coordinates were used: PrL (AP: +1.94 mm; ML: ±0.4 mm; DV: –2.3 mm) and LH (AP: –1.3 mm; ML: ±1.2 mm; DV: –5 mm). For optogenetic manipulation and fiber photometry recording, an optic fiber (200 μm; BGFLaser, Xi’an) was implanted 0.2 mm above the injection site and secured with dental cement. Subsequent experiments were performed no earlier than two weeks post-injection.

### CRISPR/Cas9-mediated knockout of *Prkab2*

To selectively knock out the *Prkab2* gene in the PrL, single-guide RNA (sgRNA) candidates targeting *Prkab2* with high specificity and efficiency were computationally identified. Three sgRNA sequences targeting the mouse *Prkab2* gene were selected:

Sequence 1: 5′-AGCTCGGAGACGTCATGTCGAGG-3′;

Sequence 2: 5′-GTTCTTTGTGGACGGACAGTGGG-3′; and

Sequence 3: 5′-ACCAGCGGATAACGGTGGGCCGG-3′.

These gRNA sequences were subsequently cloned into the pAAV2-U6-sgRNA.sp(MCS)-hSyn-mCherry plasmid to construct the vector AAV2/9-U6-sgRNA.sp(mPrkab2)×3-hSyn-mCherry. To validate the CRISPR-Cas9 construct, AAVs expressing Cas9 were bilaterally co-injected with either AAV2/9-U6-sgRNA.sp(mPrkab2)×3-hSyn-mCherry or the control AAV2/9-U6-sgRNA.sp(NC)-hSyn-mCherry into the mouse PrL. Three weeks post-injection, brain tissues were collected for western blot analysis to verify the knockout efficiency.

### Rabies virus-based monosynaptic retrograde tracing

To identify inputs to orexin neurons, we employed a monosynaptic retrograde tracing strategy using Cre-dependent helper viruses combined with the rabies virus. Briefly, a mixture of helper virus (AAV-DIO-TVA-mCherry) and AAV-DIO-RVΔG was injected into the LH (AP: –1.3 mm; ML: +1.2 mm; DV: –5 mm) of *Hcrt*-cre mice. Two weeks after this injection, a second virus, RV-EnvA-EGFP, was injected at the same site. Seven days after the second viral injection, the mice were perfused, and brain sections were subsequently imaged using a confocal microscope (Nikon ECLIPSE Ti2).

### Optogenetic and chemogenetic manipulations

Optogenetic manipulation was performed at least 14 days post-surgery. For optogenetic activation, mice received red light stimulation using a 635 nm laser set to an intensity of 3 mW, frequency of 20 Hz, and duty cycle of 20%. For optogenetic inhibition, continuous yellow light was delivered using a 590 nm laser at ~8 mW. For chemogenetic manipulation, mice were injected with either AAV2/9-hEF1a-fDIO-hM4D(Gi)-mCherry or AAV2/9-hEF1a-fDIO-mCherry into the LH and then allowed to recover for at least two weeks. Before chemogenetic inhibition, the mice were habituated to the behavioral arena for two consecutive days. Clozapine-N-oxide at a dosage of 3 mg/kg was administered intraperitoneally 30 min before behavior testing.

### Mouse behavioral tests

#### CPP behavioral test

All the mice were pre-exposed to an HFD for at least three consecutive days prior to any training or testing sessions, ensuring that the novelty-induced suppression of feeding was minimized. The CPP test was conducted in a 50 cm × 22 cm × 31 cm arena divided into two distinct chambers and a transition chamber, each separated by removable wooden walls. The two chambers featured distinct wall patterns (black-and-white striped vs solid black) and floor types (black cylindrical vs black cuboid), each measuring 20 cm × 22 cm × 31 cm. On day 1, the mice were habituated to the arena for 20 min and subsequently fasted for 16 h (from 17:00 to 09:00). On days 2–4, food was placed in the chamber with black-and-white striped walls and a black cylindrical floor (paired chamber, brighter chamber), where the mice consumed either chow or HFD for 30 min. The opposite chamber (solid black walls and a black cuboid floor), without food, was designated the unpaired chamber. After training, the mice were returned to their home cages with chow available until 17:00, after which the food was removed, and the mice were fasted again for 16 h. On day 5, the mice were allowed to move freely between the two chambers for 20 min. The time spent in the paired context (t_paired) and unpaired context (t_unpaired) was recorded using ANY-maze behavioral analysis software. The CPP score was calculated as CPP score = $\frac{({t\_paired}-{t\_\; unpaired})}{(t\_{paired}+{t\_\; unpaired})}$.

For optogenetic stimulation during the CPP test, a 635-nm laser (intensity, 3 mW; frequency, 20 Hz; and duty cycle, 20%) was activated when the mice entered the paired side and turned off upon their exit. For optogenetic inhibition, a 590-nm laser was continuously delivered at 8 mW whenever mice were present on either side of the chamber.

#### Context-paired (CTX+ ) feeding behavioral test

For the CTX food intake measurements, a training paradigm similar to that described for the CPP test (see above) was used. Briefly, during CTX– training (days 2–4), the mice were placed into the unpaired chamber (solid black walls and a black cuboid floor) for 30 min and then returned to their home cage for HFD intake. In contrast, during CTX+ training (days 2–4, following CTX– training with at least a two-week interval), mice were placed directly into a distinctly different paired chamber (black-and-white striped walls and a black cylindrical floor; brighter chamber) to consume the HFD for 30 min. On day 5, sated mice previously trained under both CTX+ and CTX– conditions were placed into their respective chambers for 30 min to measure food intake. During optogenetic stimulation experiments, food intake was measured for 20 min.

#### Real-time place preference test

ChrimsonR-expressing or mCherry-expressing mice were placed in a two-chamber apparatus (52 cm × 26 cm × 25 cm) and allowed to move freely between the two chambers. Closed-loop optogenetic stimulation (intensity, 3 mW; frequency, 20 Hz; and duty cycle, 20%) was initiated when the mice entered one side of the chamber and ceased upon their exit. The entire test lasted 20 min. The duration spent in each chamber was recorded using ANY-maze behavioral analysis software.

#### Novel object recognition test

On day 1, mice were placed in a three-chamber apparatus (60 cm × 40 cm × 22 cm) and allowed to freely explore two identical cube-shaped objects (6.5 cm × 6.5 cm × 6.5 cm) located in each side chamber for 10 min. On day 2, one of the cube-shaped objects was replaced with a novel cylindrical object (7 cm × 7 cm × 6 cm), and the mice were again allowed to explore freely for 10 min. Exploration time was defined as the duration during which the mouse’s head was located within a 2 cm radius of each object. The time spent exploring the familiar object (FO_time) and the novel object (NO_time) was automatically recorded and analyzed using ANY-maze software.

#### Contextual fear memory test

The contextual fear memory behavior test was performed in a box measuring 18 cm × 20 cm × 30.5 cm and equipped with a shock floor. On day 1, the mice were placed in the box for a 3-min habituation period and then subjected to four cycles of shock trials. Each trial consisted of a 30-s session of 80 dB white noise, which included a 0.75 mA foot shock during the final 2 s, followed by a 60-s break interval. On day 2, the mice were returned to the conditioning arena for 5 min to assess contextual fear memories. Freezing time was recorded using ANY-maze behavior analysis software.

### Fiber photometry recording and data analysis

After an AAV expressing GCaMP6s was injected into the PrL, an optic fiber was implanted 0.2 mm above the injection site and secured with dental cement. Two weeks postinjection, recordings were performed using a fiber photometry system (Thinker Biotech, Nanjing). In all the experiments, the intensity of the 470 nm light was set to 30 μW. The raw signal data were synchronized with video recordings. Fluorescence signals were recorded at a sampling rate of 100 Hz, and behavioral videos were recorded at 20 frames per second (FPS). Data analysis was performed using custom MATLAB code. The change in fluorescence (ΔF/F), calculated as (F – F0)/F0, where F0 is the mean baseline fluorescence, was used to represent neuronal activity in response to specific behaviors. For GRAB_ATP1.0_ sensor signal recordings, mice received an i.p. injection of MQ-HCl (10 mg/kg), and signal recordings were initiated 20 min post-injection. The response onset latency was defined as the first data point exceeding the baseline mean ± 2 SDs.

### Miniscope calcium imaging and data analysis

#### Mouse surgery and calcium imaging

The AAV injection procedure was performed as described above. Following the injection, a GRIN lens (CLHS100GFT003, GOFOTON, 1.0 mm outer diameter, and 3.71 mm length) was implanted ~0.2 mm above the injection site and secured with dental cement. After implantation, the mice were administered ceftriaxone sodium (80 mg/kg, i.p.) for three consecutive days and were singly housed. Three weeks later, a baseplate was installed on the mice for which neuronal morphology was clearly visible. The mice were then allowed to recover for one week before the imaging experiments. Calcium fluorescence videos were acquired at 30 FPS using the UCLA Miniscope V4, and behavioral videos were simultaneously recorded using Miniscope-DAQ-QT-Software (https://github.com/Aharoni-Lab/Miniscope-DAQ-QT-Software).

#### Calcium signal extraction

For calcium imaging analysis, calcium fluorescence videos were first integrated using the Miniscope Analysis package (https://github.com/etterguillaμme/MiniscopeAnalysis). The integrated videos were subsequently used for calcium signal extraction. This process involved motion correction (https://github.com/flatironinstitute/NoRMCorre)^88^ and cellular signal isolation using constrained non-negative matrix factorization (CNMF-E, https://github.com/zhoupc/CNMF_E)^89^. The ∆F/F calcium traces of individual cells were then z-scored (presented in units of standard deviation) prior to further analysis. To ensure accuracy in neuronal signal extraction, all raw neurons identified by the CNMF-E package were manually inspected, allowing for the exclusion of regions of interest that either lacked intact neuron somas or contained excessive imaging boundaries.

#### Analysis of event-activated neurons

To analyze neurons activated during specific events, calcium imaging signal data corresponding to these events were extracted on the basis of synchronously recorded behavior videos. This included 10 s of data before and 10 s after event onset, encompassing a total of 600 frames. The data of 5 s before event onset were used as the baseline, while the data of 5 s following onset were designated as event data. For the analysis, both baseline and event data from all extracted cells were first subjected to a permutation test to identify activated cells^90^. Neurons were defined as event-activated if their average z-scored ∆F/F exceeded 0.2 after the permutation test.

To identify neurons specifically activated in corresponding events, we used the Cell registration analysis package (https://github.com/zivlab/CellReg)^91^ to extract all neuronal calcium signals across two events. The activated cell data from both events were extracted, and the average z-scored ∆F/F for each event was calculated. We then computed the SRI using the formula (A – B)/(A + B)^92^, where A and B represent the average z-scored ∆F/F of the response periods in events A and B, respectively. Cells with an SRI greater than 0.2 were considered specifically activated in event A, whereas those with an SRI less than –0.2 were identified as specifically activated in event B. Cells with an SRI ranging from –0.2 and 0.2 were classified as activated in both events.

#### Identification of pre-cells and post-cells

To examine the interaction of neural responses to environmental contexts and HFD consumption, we analyzed the activity of neurons responsive to paired contexts. In the pre-cell analysis, we used data from –10 s to –5 s before HFD feeding bouts as baseline data, while data from –5 s to 0 s before feeding were treated as event data. We then performed a permutation test on these datasets. Neurons whose maximum z-scored ∆F/F was greater than 0.2 in the event data were classified as pre-cells. For post-cell analysis, data from –5 s to 0 s before HFD feeding were used as baseline data, and data from 0 s to 5 s after feeding were considered event data. Similarly, a permutation test was performed, and neurons with a maximum z-scored ∆F/F exceeding 0.2 in the event data were classified as post-cells.

#### Binary decoding between different types of events

We used a linear binary SVM to predict behaviors on the basis of calcium signals activated in two different events (Supplementary Fig. S2b, c, e, f). First, we prepared a dataset comprising the neuronal activity array of cells activated in these events, along with the corresponding behavioral labels. Ca^2+^ activity was organized into an N × M × T dimensional matrix, where N represents the number of activated cells in the two events, M is the number of trials and T refers to the neural activity recorded in each frame. The dataset was subsequently used to train the SVM for behavior prediction (10-fold cross-validation). To generate shuffled data as a control, we randomly permuted the binary behavior table and performed the same decoding. Decoder performance was calculated as the percentage of correctly predicted labels out of the total number of labels. The cumulative distribution function (CDF) of the decoder performance was generated on the basis of the prediction data obtained from each fold.

For the decoder projections^53^ shown in Figs. 2l and 4o, single-behavior decoders were constructed using samples of neural activity from neurons activated during specific behaviors, along with baseline activity samples taken from a 5-s period prior to CTX + HFD feeding or entries into CTX+ paired contexts. To generate a continuous projection over an interval of test data, the entire set of neural activity data was split into two consecutive 50% segments. The first 50% of the segments were used for SVM analysis to establish the decoder hyperplane. The remaining 50% of the data were subsequently projected onto the component normal to this plane to generate a continuous projection. Decoder predictions exceeding the prediction threshold are visually indicated with shaded areas.

For the behavior decoding time courses^53^ used in discriminating the two behaviors shown in Figs. 2g, k, 4n and Supplementary Fig. S4m, the SVM decoders were trained and tested using population activity from activated neurons in two events at different time points relative to the actual onset of behavior. At each specified time point, the population activity was calculated as the average activity of the 1-s data following that point. The performance of these decoders was assessed using 5-fold cross-validation. The control was trained using shuffled neural activity and behavior labels.

### RNA sequencing and data analysis

For bulk RNA-seq on fed and fasted mice, food was withheld from the fasted group for 24 h, while the fed group had ad libitum access to food. For bulk RNA-seq on CTX– and CTX+ mice, following the conditioning training described above, the CTX– and CTX+ mice were re-exposed to their respective training contexts for 30 min. Subsequently, bilateral PrL brain tissues were dissected for total RNA isolation. RNA quality was assessed via gel electrophoresis and with a Qubit fluorometer (Thermo, Waltham, MA, USA). For RNA sequencing, 1 μg of total RNA was used for library construction. Sequencing libraries were prepared using the VAHTS™ Stranded mRNA-seq Library Prep Kit for Illumina^®^. The libraries were sequenced as 150 bp paired-end reads on an Illumina NovaSeq 6000. The gene counts of all the samples were used to perform PCA and GSEA with the gseGO code in R Studio (2023).

### Statistical analysis

The Kolmogorov–Smirnov test was applied to determine whether the data followed a normal distribution. For datasets exhibiting a normal distribution, a *t*-test was used for pairwise comparisons. For multiple comparisons, one-way ANOVA followed by Tukey’s post hoc comparison was conducted. In cases where the data did not follow a normal distribution, the non-parametric Mann–Whitney test was used. For analyses involving two-way ANOVA, Sidak’s multiple comparison post hoc test was applied. The data are presented as mean ± SEM. The significance levels are denoted as follows: *P < 0.05, **P < 0.01, ***P < 0.001, and ****P < 0.0001. All the statistical analyses were performed using Prism software.

## Supplementary information

Supplementary information

## Data Availability

All data and code generated in this study are available upon reasonable request by contacting the corresponding author Zhi-Xiang Xu.

## Code availability

All data and code generated in this study are available upon reasonable request by contacting the corresponding author Zhi-Xiang Xu.
