# Supplementary material for: Prefrontal cortex-to-hypothalamic outputs orchestrate cue-potentiated palatable food consumption via AMPKβ2 signaling
Source: Cell Discov. 2026 Jan 6;12:2. doi: 10.1038/s41421-025-00857-2 (PMC12775431; doi:10.1038/s41421-025-00857-2)
Supplement: Supplementary file 1 — Supplementary information [file 41421_2025_857_MOESM1_ESM.pdf]

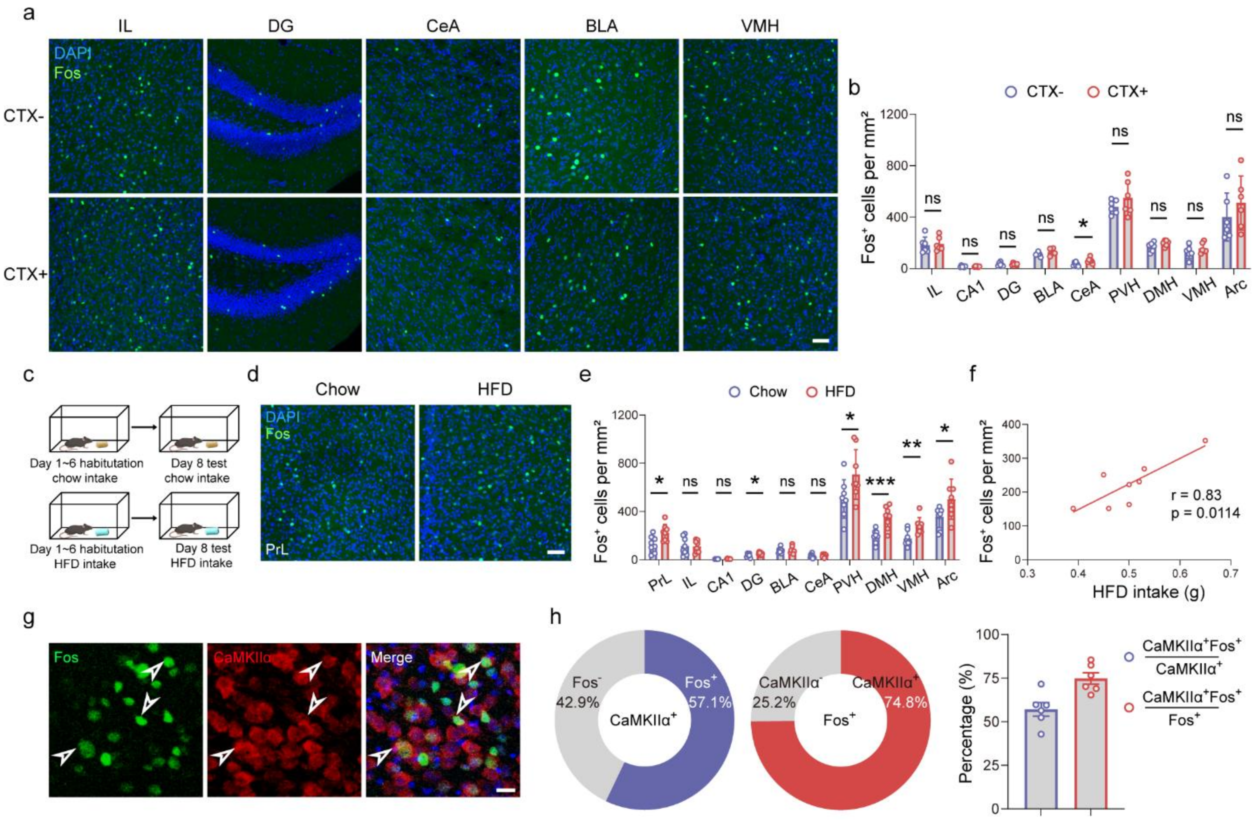

Supplementary Fig S1. Fos expression in multiple brain regions during HFD consumption and exposure to HFD-paired environmental contexts

(a) Representative images showing Fos$^+$ cells in the IL, DG, CeA, BLA and VMH after mice were exposed to CTX+ or CTX- contexts. Scale bar, 50 μm.

(b) Quantification of Fos$^+$ cells in the IL, CA1, DG, BLA, CeA, PVH, DMH, VMH, and Arc after mice were exposed to CTX+ or CTX- contexts. *$p < 0.05$, ns not significant, $p > 0.05$. 6 mice per group.

(c) Schematic for chow and HFD intake measurement.

(d) Representative images showing Fos$^+$ cells in the PrL after chow or HFD feeding. Scale bar, 50 μm

(e) Quantification of Fos^+ cells in the PrL, IL, CA1, DG, BLA, CeA, PVH, DMH, VMH, and Arc after chow or HFD feeding. n = 8 mice per group, unpaired *t*-test, *$p < 0.05$, ns not significant, $p > 0.05$.

(f) Correlation analysis between Fos^+ cell numbers in the PrL and the amount of HFD intake. Pearson correlation analysis, $p = 0.011$. n = 8 mice.

(g) Representative images showing colocalization of Fos and CaMKIIα after HFD consumption. Scale bar, 20 μm.

(h) Percentage of $CaMKII\alpha^+Fos^+$ cells among total $CaMKII\alpha^+$ cells or total Fos^+ cells in the PrL after HFD intake. n = 6 mice.

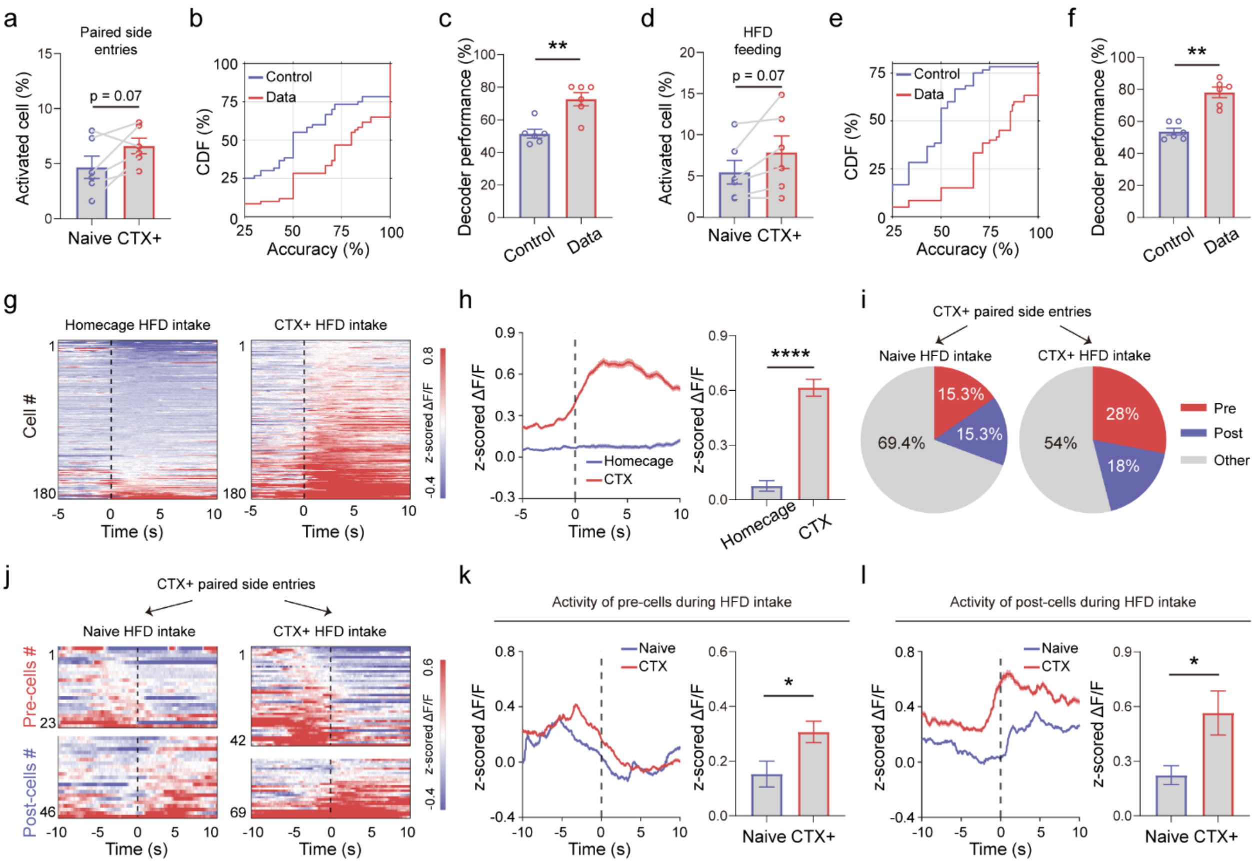

Supplementary Fig S2. Neural dynamics of PrL$^{CaMKII\alpha+}$ neurons in response to HFD consumption and the paired environmental contexts.

(a) Percentage of neurons activated upon entries into the paired chamber before (naive) and after (CTX+) conditioning training. Paired t-test, p = 0.0751.

(b) Cumulative distribution of decoding accuracy of the SVM decoder used to discriminate between neutral (naive) and paired (CTX+) chamber entries.

(c) Decoder performance in distinguishing between neutral (naive) and paired (CTX+) chambers. Paired t-test, p = 0.0075, n = 6 mice.

(d) Percentage of neurons activated during HFD intake in neutral (naive) and paired (CTX+) chambers. Paired t-test, p = 0.0693. n = 6 mice.

(e) Cumulative distribution of decoding accuracy of the SVM decoder to discriminate between HFD intake in neutral (naive) and paired (CTX+) chamber.

(f) Decoder performance in distinguishing between HFD intake in neutral (naive) and paired (CTX+) chambers. Paired t-test, $p = 0.0039$, $n = 6$ mice.

(g) Heatmap showing the activity of $PrL^{CaMKII\alpha+}$ neurons during HFD intake in the paired chamber and corresponding neuronal activity during HFD intake in the homecage.

(h) Z-scored activity of $PrL^{CaMKII\alpha+}$ neurons during HFD intake in the paired chamber and the corresponding neuronal activity during HFD intake in the homecage. Bar graphs show the mean z-scored $\Delta F/F$ from 0-5 s during HFD intake bouts. Wilcoxon matched-pairs signed rank test, $p < 0.0001$.

(i) Proportion of defined 'pre' and 'post' cells among neurons activated in the paired chamber.

(j) Heatmap of neural activity of defined 'pre' and 'post' cells during HFD feeding in the neutral (naive) and paired (CTX+) chambers. The dark dashed line is aligned with the onset of HFD intake.

(k) Z-scored responses of 'pre' cells during HFD feeding in the neutral (naive) and paired (CTX+) chambers. The bar graph quantifies the mean z-scored $\Delta F/F$ from -5 to 0 seconds. $n = 23$ cells for HFD intake in neutral chamber and $n = 42$ cells for HFD intake in the paired chamber. Mann-Whitney test, $^*p < 0.05$.

(l) Z-scored responses of 'post' cells during HFD intake in the neutral (naive) and paired (CTX+) chambers. The bar graph quantifies the mean z-scored $\Delta F/F$ from 0 to 5 seconds. $n = 23$ cells for HFD intake in neutral chamber and $n = 27$ cells for HFD intake in the paired chamber. Mann-Whitney test, $^*p < 0.05$.

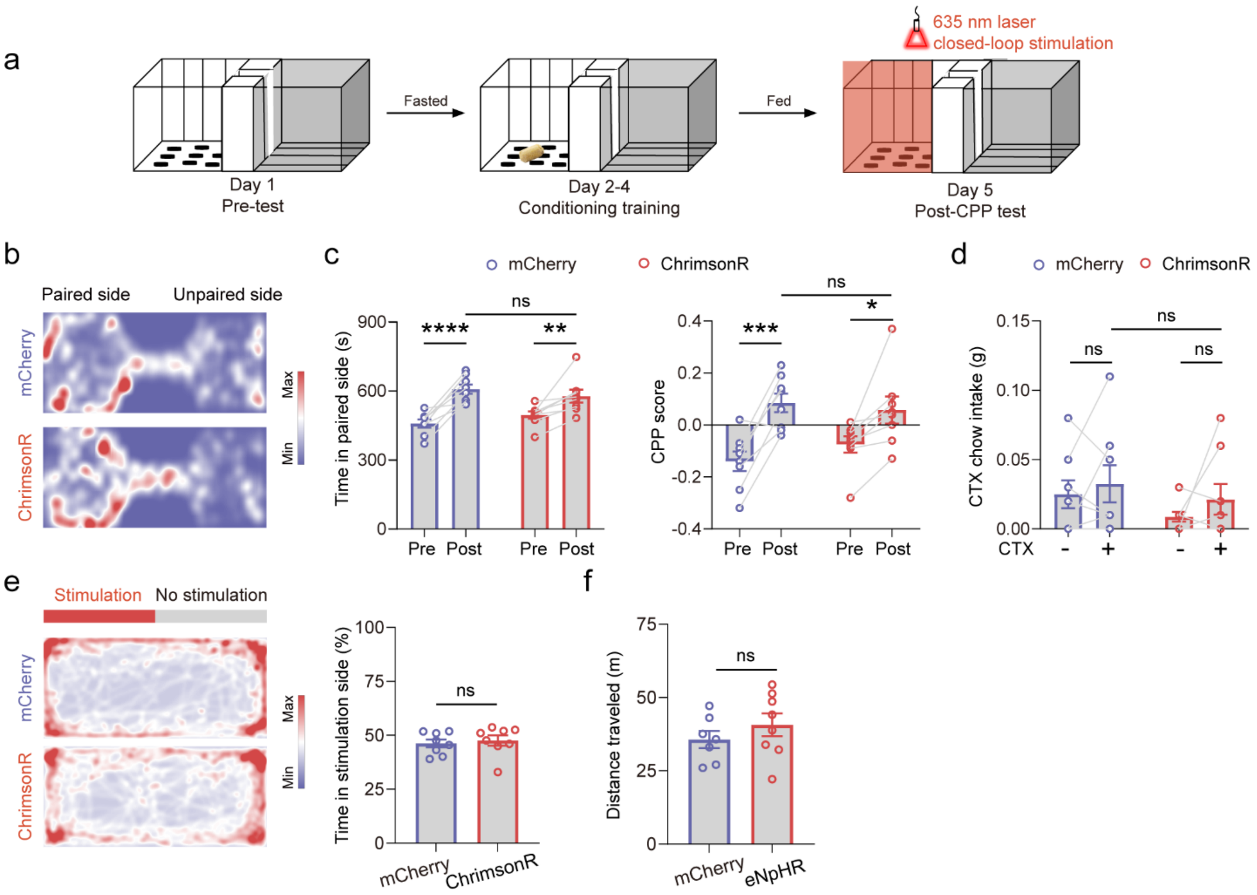

Supplementary Fig S3. Optogenetic activation of $\mathrm{PrL}^{\mathrm{CaMKII\alpha+}}$ neurons in paired context did not alter chow intake.

(a) Behavioral schematic for examining place preference for chow-paired environmental contexts following conditioning training with closed-loop optogenetic stimulation of $\mathrm{PrL}^{\mathrm{CaMKII\alpha+}}$ neurons.

(b) Heatmap representing the position of mice in the experimental arena during the CPP test for mice expressing mCherry or ChrimsonR in the PrL.

(c) Time spent on the paired side before and after paired training, and CPP scores of mice expressing mCherry or ChrimsonR. Repeated-measures two-way ANOVA followed by Sidak's multiple comparison post hoc test. $F_{(1, 14)} = 46.87$, $p < 0.0001$ for time; $F_{(1, 14)} = 37.13$, $p < 0.0001$ for CPP scores. mCherry, 8 mice, ChrimsonR, 8 mice.

(d) Chow intake in the CTX- and CTX+ chambers measured in mice expressing mCherry or ChrimsonR in the PrL. Repeated-measures two-way ANOVA followed by Sidak's multiple comparison post hoc test. $F_{(1, 14)} = 1.358$, $p = 0.2634$ for contexts; mCherry, 8 mice; ChrimsonR, 8 mice.

(e) Heatmap showing locomotion trace in mice expressing mCherry or ChrimsonR in RTPP test. Bars represent the percentage of time spent on the stimulation side in both groups. n = 8 mice per group, ns not significant, $p > 0.05$.

(f) Total distance traveled in mice expressing mCherry or eNpHR in the CPP test. n = 7 for mCherry mice and 8 for eNpHR mice, ns not significant, $p > 0.05$.

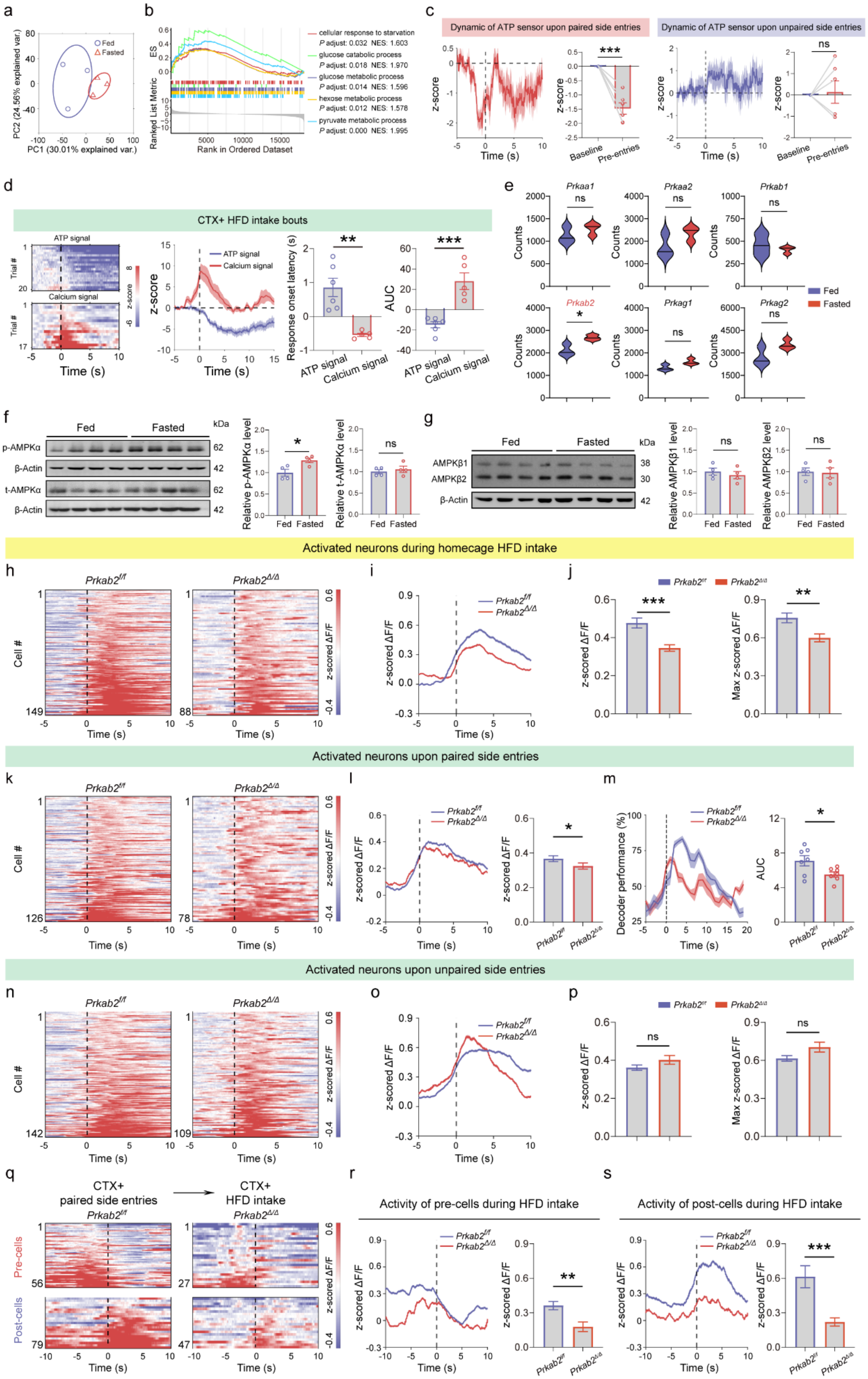
a
b
c
Dynamic of ATP sensor upon paired side entries
Dynamic of ATP sensor upon unpaired side entries
d
CTX+ HFD intake bouts
ATP signal
Calcium signal
e
Fed
Fasted
Prkaa1
Prkaa2
Prkab1
Prkab2
Prkag1
Prkag2
f
g
Activated neurons during homecage HFD intake
h
i
j
Activated neurons upon paired side entries
k
l
m
Activated neurons upon unpaired side entries
n
o
p
q
CTX+ paired side entries
CTX+ HFD intake
Pre-cells
Post-cells
r
Activity of pre-cells during HFD intake
s
Activity of post-cells during HFD intake

Supplementary Fig S4. Depletion of AMPKβ2 in the PrL abolishes cue-potentiated HFD overconsumption.

(a) Principal component analysis of gene expression in the PrL from fed and fasted mice.

(b) GSEA plots showing significantly enriched gene signatures in the PrL of fed and fasted mice.

(c) Z-scored responses of the ATP fluorescent sensor during paired side and unpaired side entries. Baseline and pre-entries quantified the mean data of −5 to −3 s and −2 to 0 s prior to entries, respectively. n = 6 mice, paired t-test, p = 0.0007 (left) and p = 0.80 (right).

(d) Heatmap and averaged response of ATP and calcium signals aligned to the onset of HFD intake in the conditioned context (CTX+). Vertical dashed lines indicate biting onset. Quantification shows a significant difference in response onset latency and area under the curve (AUC) between ATP signals and calcium signals (unpaired t-test; response onset latency, p = 0.001; AUC, p = 0.0005). n = 6 mice for ATP signaling, and n = 5 mice for calcium signaling.

(e) Expression of Prkaa1, Prkaa2, Prkab1, Prkab2, Prkag1, Prkag2 in the PrL of fed and fasted mice. *p < 0.05; ns not significant, p > 0.05, n = 3 mice per condition.

(f) Immunoblotting of p-AMPKα and t-AMPKα in the LH of sated and fasted mice. Unpaired t-test, *p < 0.05, ns not significant, p > 0.05, n = 4 mice per group.

(g) Expression levels of AMPKβ1 and AMPKβ2 in the LH of sated and fasted mice. ns not significant, p > 0.05, n = 4 mice per group.

(h) Heatmap of neural activity of activated neurons during HFD consumption in the homecage for prkab2$^{fl/fl}$ and prkab2$^{\Delta/\Delta}$ mice. The dark dashed line is aligned with the onset of HFD intake. prkab2$^{fl/fl}$ mice, 149 activated neurons out of a total of 1829 neurons in 7 mice; prkab2$^{\Delta/\Delta}$ mice, 88 activated neurons out of a total of 1651 neurons in 8 mice.

(i) Response of activated neurons during HFD consumption in the homecage for prkab2$^{fl/fl}$ and prkab2$^{\Delta/\Delta}$ mice.

(j) Mean and maximal z-scored ΔF/F of activated cells during the first 0-5 s at the onset of homecage HFD feeding in prkab2$^{fl/fl}$ and prkab2$^{\Delta/\Delta}$ mice. Mann-Whitney test, ***$p < 0.001$.

(k) Heatmap of neural activity of activated neurons upon entry into the paired chamber in prkab2$^{fl/fl}$ and prkab2$^{\Delta/\Delta}$ mice. The dark dashed line is aligned with the onset of entry into the paired chambers. prkab2$^{fl/fl}$ mice, 126 activated neurons out of a total of 1938 neurons in 7 mice; prkab2$^{\Delta/\Delta}$ mice, 78 activated neurons out of a total of 1458 neurons in 8 mice.

(l) Average response of activated cells upon entry into paired chambers in prkab2$^{fl/fl}$ and prkab2$^{\Delta/\Delta}$ mice. Bar graphs represent the mean z-scored ΔF/F during 0-5 s after entry. Mann-Whitney test, $p = 0.0435$.

(m) SVM decoder accuracy relative to behavior onset in discriminating between HFD-paired and unpaired chambers in prkab2$^{fl/fl}$ and prkab2$^{\Delta/\Delta}$ mice. The AUC of the decoder performance during 0-10 s was computed. Time 0 is aligned with chamber entries. Unpaired t-test, *$p < 0.05$, $n = 7$ mice per condition.

(n) Heatmap of neural activity of activated neurons upon entry into the unpaired chamber in prkab2$^{fl/fl}$ and prkab2$^{\Delta/\Delta}$ mice. The dark dashed line is aligned with the onset of entries into the unpaired chambers. prkab2$^{fl/fl}$ mice, 142 activated neurons out of a total of 1938 neurons in 7 mice; prkab2$^{\Delta/\Delta}$ mice, 109 activated neurons out of a total of 1458 neurons in 8 mice.

(o) Response of activated neurons upon entries into the unpaired chamber in prkab2$^{fl/fl}$ and prkab2$^{\Delta/\Delta}$ mice.

(p) Mean and maximal ΔF/F of activated cells upon entry into the unpaired chamber. ns not significant, $p > 0.05$.

(q) Heatmap of neural activity of defined 'pre' and 'post' cells during HFD consummatory bouts in the paired chamber for prkab2$^{fl/fl}$ and prkab2$^{\Delta/\Delta}$ mice. The dark dashed line is aligned with the onset of HFD intake, and the red dashed line is aligned to 5 s before HFD feeding.

(r) z-scored responses of 'pre' cells during HFD feeding in the paired (CTX+) chamber of prkab2$^{fl/fl}$ and prkab2$^{\Delta/\Delta}$ mice. The bar graph quantifies the mean z-scored ΔF/F from -5 to 0 seconds. n = 56 pre-cells for prkab2$^{fl/fl}$ mice and 27 pre-cells for prkab2$^{\Delta/\Delta}$ mice. Mann-Whitney test. **$p < 0.01$.

(s) z-scored responses of 'post' cells during HFD intake in the paired (CTX+) chamber of prkab2$^{fl/fl}$ and prkab2$^{\Delta/\Delta}$ mice. The bar graph quantifies the mean z-scored ΔF/F from 0 to 5 seconds. n = 23 post-cells for prkab2$^{fl/fl}$ mice and 20 post-cells for prkab2$^{\Delta/\Delta}$ mice. Mann-Whitney test. ***$p < 0.001$

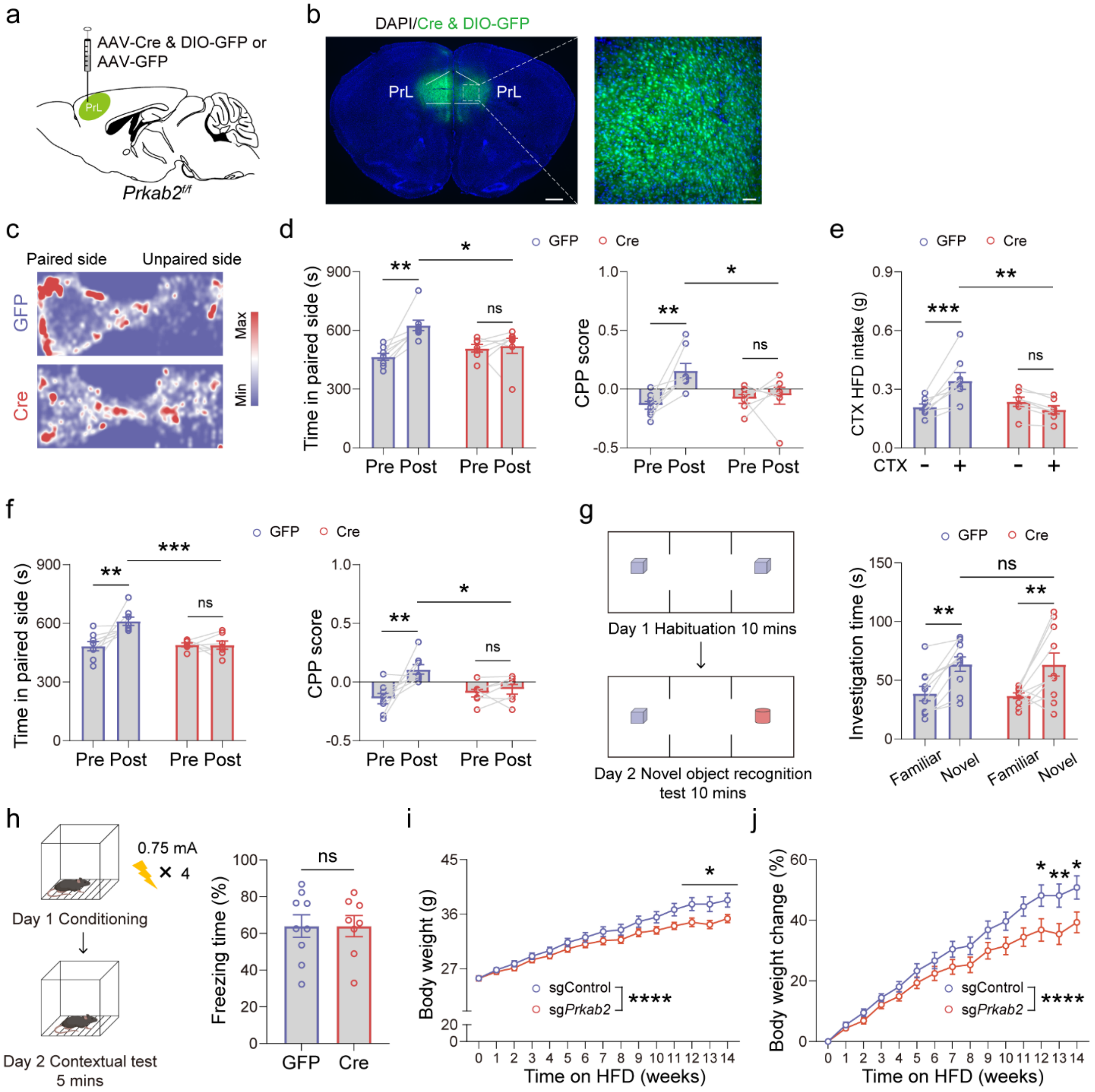

Supplementary Fig S5. PrL AMPKβ2 knockout impairs conditioned preference and cue-potentiated palatable food intake, without affecting novel object recognition or fear conditioning.

(a) Experimental strategy for viral-mediated deletion of *Prkab2* in the PrL using the Cre–loxP system.

(b) Representative images showing GFP expression in the PrL (DAPI, blue; GFP, green). Scale bars, 500 μm (left) and 100 μm (right).

(c) Heatmaps of mouse position during the CPP test for mice expressing GFP or Cre + GFP in the PrL after HFD paired training.

(d) Time spent on the paired side (left) and CPP score (right) before (Pre) and after (Post) HFD paired training in GFP vs Cre + GFP mice. Repeated-measures two-way ANOVA followed by Sidak's multiple comparison post hoc test. $F_{(1, 13)} = 8.233$, $p = 0.013$ for interaction in time comparison; $F_{(1, 13)} = 5.589$, $p = 0.034$ for interaction in CPP scores comparison. GFP, 8 mice; Cre, 7 mice.

(e) HFD intake in the CTX+ and CTX− chambers in GFP vs Cre + GFP mice after HFD paired training. Repeated measures two way ANOVA with Sidak's multiple comparisons post hoc test; Interaction: $F_{(1,13)}=22.51$, $p=0.0004$. GFP, n=8; Cre, n=7.

(f) Time on the paired side (left) and CPP score (right) before and after HSD paired training in GFP vs Cre + GFP mice. Repeated-measures two-way ANOVA followed by Sidak's multiple comparison post hoc test. $F_{(1, 13)} = 14.88$, $p = 0.002$ for virus; $F_{(1, 13)} = 5.946$, $p = 0.029$ for interaction in CPP scores comparison. GFP, 8 mice; Cre, 7 mice.

(g) Schematic of the novel object recognition (NOR) test (left). Investigation time for familiar vs novel objects (right) in GFP vs Cre + GFP mice. Repeated-measures two-way ANOVA followed by Sidak's multiple comparison post hoc test. $F_{(1, 18)} = 0.0192$, $p = 0.89$ for virus. GFP, 10 mice; Cre, 10 mice.

(h) Schematic of contextual fear conditioning (left). Freezing time during memory retrieval (right) in GFP vs Cre + GFP mice. Unpaired two tailed t test; ns, $p>0.05$. GFP, n=9; Cre, n=8.

(i) Body weight of sgControl and sgPrkab2 mice fed an HFD over 14 weeks. Two way ANOVA with Sidak's multiple comparisons post hoc test; $F_{(1,300)}$=37.08, p<0.0001; significance indicated on the plot. n=11 mice per group.

(j) Body weight gain in sgControl and sgPrkab2 mice fed an HFD. Two way ANOVA with Sidak's multiple comparisons post hoc test; $F_{(1,300)}$=42.07, p<0.0001; * p<0.05, ** p<0.01, ****p<0.0001. n=11 mice per group.

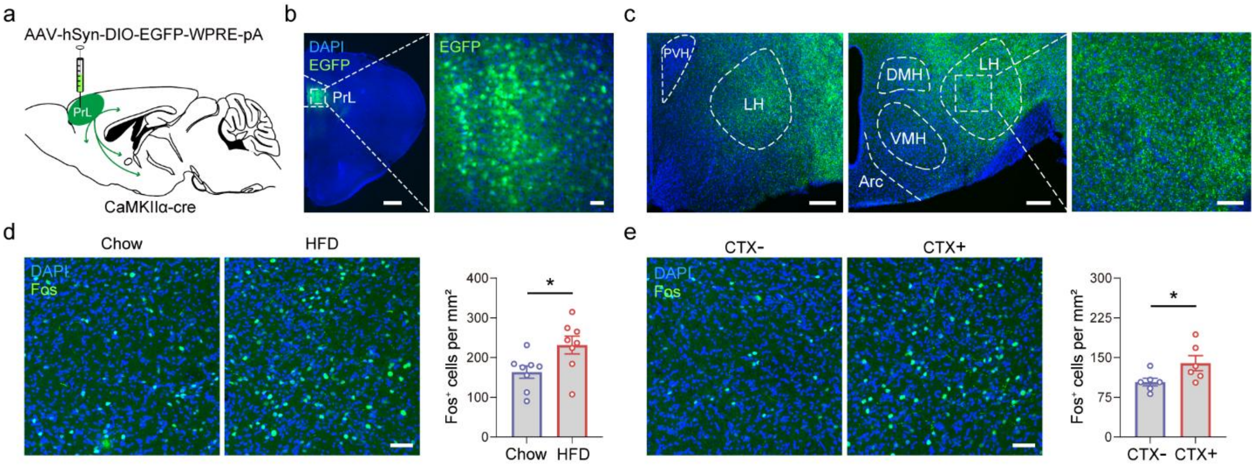

Supplementary Fig S6. Distribution of axon terminals of PrL neurons in the hypothalamus and activation of LH neurons by HFD consumption and HFD-paired environmental contexts.

(a) Diagram illustrating virus injection in the PrL to trace axonal outputs to subregions of the hypothalamus.

(b) Representative images showing the AAV injection site in the PrL. Scale bars, 500 μm (left) and 100 μm (right), respectively.

(c) Representative images showing the axon terminals of PrL neurons projecting into hypothalamic areas. Scale bars, 200 μm (left), 200 μm (middle) and 100 μm (right), respectively.

(d) Fos^{+} cells in the LH after chow or HFD feeding. Scale bar, 50 μm. 8 mice per group, unpaired t-test, p = 0.0259.

(e) Fos$^+$ cells in the LH after mice were exposed to CTX+ or CTX- contexts. Scale bar, 50 μm; 6 mice per group, unpaired t-test, p = 0.0494.

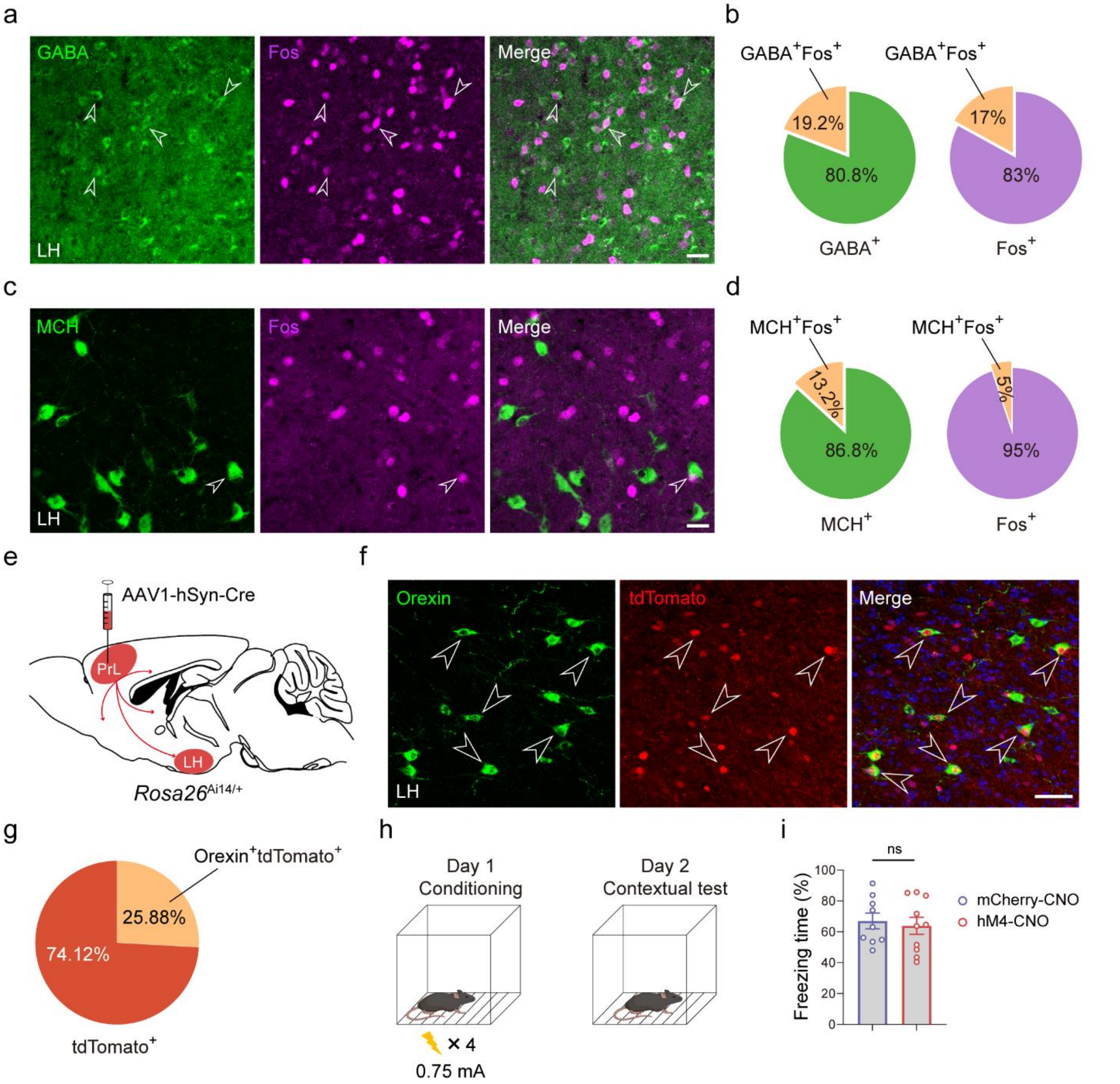

Supplementary Fig S7. Cellular mechanisms through which the PrL→LH circuit regulates cue-potentiated HFD overconsumption.

(a) Representative images showing the colocalization of Fos and GABA in the LH following stimulation of PrL-LH pathway. Scale bar, 25 μm.

(b) Percentage of $GABA^{+}Fos^{+}$ cells among total $GABA^{+}$ cells and among total Fos^{+} cells. n = 5 mice.

(c) Representative images showing the colocalization of Fos and MCH in the LH following stimulation of the PrL-LH pathway. Scale bar, 25 μm.

(d) Percentage of $MCH^{+}Fos^{+}$ cells among total MCH^{+} cells and among total Fos^{+} cells. n = 4 mice.

(e) Virus injection strategy for anterograde transsynaptic labeling of the PrL-LH pathway in $Rosa26^{Ai14/+}$ mice.

(f) Colocalization of orexin with transsynaptic labeled $tdTomato^{+}$ neurons from the PrL. Scale bar, 50 μm.

(g) Percentage of $Orexin^{+}tdTomato^{+}$ neurons among total $tdTomato^{+}$ cells. n = 3 mice.

(h) Schematic of the contextual fear conditioning behavioral test.

(i) Freezing time in mice expressing mCherry or hM4Di following CNO injection during contextual fear memory retrieval. Unpaired t-test, ns not significant, $p > 0.05$, mCherry, 9 mice; hM4Di, 10 mice.
